# ﻿Morphological and phylogenetic analyses reveal eight novel species of *Pestalotiopsis* (Sporocadaceae, Amphisphaeriales) from southern China

**DOI:** 10.3897/mycokeys.109.131000

**Published:** 2024-10-09

**Authors:** Xing-Xing Luo, Ming-Gen Liao, Kai Zhang, Rafael F. Castañeda-Ruíz, Jian Ma, Zhao-Huan Xu

**Affiliations:** 1 College of Agronomy, Jiangxi Agricultural University, Nanchang, Jiangxi 330045, China; 2 College of Forestry Engineering, Shandong Agriculture and Engineering University, Jinan 250100, China; 3 Instituto de Investigaciones de Sanidad Vegetal, Calle 110 No. 514 e/5ta B y 5ta F, Playa, La Habana 11600, Cuba; 4 Jiangxi Key Laboratory for Excavation and Utilization of Agricultural Microorganisms, Jiangxi Agricultural University, Nanchang, Jiangxi 330045, China

**Keywords:** Asexual Ascomycota, molecular phylogeny, new species, Sordariomycetes, taxonomy

## Abstract

Plants play an important role in maintaining the ecological balance of the biosphere, but often suffer from pathogenic fungi during growth. During our continuing mycological surveys of plant pathogens from terrestrial plants in Jiangxi and Yunnan provinces, China, 24 strains of *Pestalotiopsis* isolated from diseased and healthy tissues of plant leaves represented eight new species, viz. *P.alpinicola*, *P.camelliicola*, *P.cyclosora*, *P.eriobotryae*, *P.gardeniae*, *P.hederae*, *P.machiliana* and *P.mangifericola*. Multi-locus (ITS, *tef1-α* and *tub2*) phylogenetic analyses were performed using maximum-likelihood and Bayesian inference to reveal their taxonomic placement within *Pestalotiopsis*. Both molecular phylogenetic analyses and morphological comparisons supported them as eight independent taxa within *Pestalotiopsis*. Illustrations and descriptions of these eight taxa were provided, in conjunction with comparisons with closely related taxa in the genus. This work highlights the large potential for new fungal species associated with diseased plant leaves.

## ﻿Introduction

Fungi are widely distributed and highly diverse in nature, forming large and complex ecosystems that play crucial roles in many biological processes (Schimann et al. 2017). Current estimates of fungal diversity are highly uncertain, ranging from 1.5 to 12 million species (Wu et al. 2019; Hyde et al. 2021; Bhunjun et al. 2022). The abundance of fungi remains to be unexplored, and only 10% of fungi were currently described (Hyde et al. 2021), but most species lack the molecular data before the advent of Sanger sequencing. In recent years, with the development of molecular techniques, the DNA-based species delimitation techniques are maturing gradually and have become an important approach to evaluate the fungal phylogenetic relationships and taxonomic placements in the study of modern fungal classification.

*Pestalotiopsis* Steyaert is a species-rich asexual genus with conidial appendages in the family Sporocadaceae Corda (Barr 1975, 1990; Kang et al. 1998, 1999), which was originally introduced to accommodate those *Pestalotia*-like species that have 5-celled conidia rather than 6-celled conidia (Steyaert 1949), and such a morphological distinction was subsequently supported by further evidence of the electronic microscopy (Guba 1961; Steyaert 1963; Griffiths and Swart 1974a,b; Sutton 1980). For *Pestalotiopsis* species, the traditional taxonomy of delineating interspecific relationships is mainly based on morphological characteristics, and most species are distinguished by conidial dimensions (Maharachchikumbura et al. 2011). Based on morphological and multi-locus phylogenetic analyses, Maharachchikumbura et al. (2014) proposed two segregated anamorphic genera from *Pestalotiopsis*, namely *Neopestalotiopsis* Maharachch., K.D. Hyde & Crous and *Pseudopestalotiopsis* Maharachch., K.D. Hyde & Crous to accommodate *Pestalotiopsis* species. *Neopestalotiopsis* is distinguished from *Pestalotiopsis* and *Pseudopestalotiopsis* by its multicolored median cells, and *Pseudopestalotiopsis* has three darker median cells compared to *Pestalotiopsis*.

To data, about 437 epithets for *Pestalotiopsis* have been listed in Index Fungorum (Index Fungorum 2024). Members of the genus are widely distributed in tropical and temperate regions as endophytes, plant pathogens or saprobes (Bate-Smith and Metcalfe 1957; Maharachchikumbura et al. 2012, 2014), but occasionally, some species of *Pestalotiopsis* have been reported as mycoparasites, human and insect pathogens (Lv et al. 2011; Monden et al. 2013; Xie et al. 2014; Li et al. 2017). The genus *Pestalotiopsis* has received considerable attention in recent years, and more research on its species diversity is still needed.

China is considered an important Asian reservoir of biodiversity by the Convention on Biological Diversity. Its rich vegetation and varied climatic regimes create a very wide range of habitats favoring the development of various microbial species. During ongoing mycological surveys of plant pathogens from terrestrial plants in Jiangxi and Yunnan provinces, 24 *Pestalotiopsis* strains isolated from diseased plant leaves are obtained. Based on morphological and multi-locus (ITS, *tef1-α* and *tub2*) phylogenetic analyses, eight *Pestalotiopsis* species were proposed as new to science in the present study.

## ﻿Materials and methods

### ﻿Sample collection, fungal isolation and morphological characterization

Samples of plant disease leaves were collected from different habitats in Yunnan and Jiangxi provinces, China, labeled and returned to the laboratory in Ziploc™ bags. The tissue isolation method was used for the isolation and identification of pathogenic fungi in this study (Gao et al. 2014). The fresh leaves were washed with running water to remove dirt and dust, then tissue pieces of junction from the diseased and healthy parts of plant leaves were cut into small pieces (5 × 5 mm). The tissue pieces were surface-sterilized with 75% ethanol for 1 min and 5% sodium hypochlorite (NaClO) for 45 s, then washed 3 times with sterile distilled water for 20 s each time, placed on sterilized filter paper to dry out the water, the tissue pieces were transferred to the potato dextrose agar (PDA, 200 g potato, 20 g glucose, 20 g agar, and 1000 mL water) plates and incubated at 25 °C in darkness until spores germinated, and the hyphal tip of individual colonies were transferred to fresh PDA plates to obtain a pure culture for further study. All fungal strains were stored in 10% sterilized glycerin at 4 °C for further studies. Cultural characteristics were observed and recorded after 7 days. Morphological characteristics were examined using an Olympus BX 53 compound microscope and photographed using the Olympus DP 27 digital camera (Olympus Optical Co., Tokyo, Japan) with a 60 × objective at the same background color and scale, and the conidia were randomly selected for measurement. The studied specimens and cultures were deposited in the Herbarium of Jiangxi Agricultural University, Plant Pathology, Nanchang, China (HJAUP). The names of the new taxa were registered in Index Fungorum (http://www.indexfungorum.org/Names/Names.asp).

### ﻿DNA extraction, PCR amplification and sequencing

When the single colonies on PDA were grown for 7 days, approximately 500 mg of fresh fungal mycelia were scraped for the total genomic DNA extraction using the Solarbio Fungi Genomic DNA Extraction Kit (Beijing Solarbio Science & Technology Co., Ltd., Beijing, China) following the manufacturer’s protocol. To confirm the species, the regions (ITS, *tef1-α* and *tub2*) of all fungal isolates were sequenced. A portion of the internal transcribed spacer (ITS), translation elongation factor 1- alpha gene (*tef1-α*) and β-tubulin (*tub2*) loci were amplified using primers pairs ITS5/ITS4 (White et al. 1990), EF1-728F/EF1-986R (Carbone and Kohn 1999) and Bt2a/Bt2b (Glass and Donaldson 1995), respectively. The corresponding primer pairs and PCR processes are listed in Table 1. The PCR mixture consisted of 10 µL Power Taq PCR Master Mix, 7.4 µL double-distilled water (ddH_2_O), 0.8 µL of each primer, and 1 µL template DNA were made up to the final volume of 20 µL. The PCR amplification products were checked via electrophoresis in 1% agarose gels and stained with ethidium bromide. Purification and sequencing of PCR products were carried out at Beijing Tsingke Biotechnology Co., Ltd., Beijing, China. The newly obtained sequences were deposited in NCBI GenBank (www.ncbi.nlm.nih.gov, accessed on 28 June Table 2).

**Table 1.** Primers and PCR program used in this study.

Locus	Primers		PCR Program
	Name	Sequence 5′–3′	
ITS	ITS5	GGAAGTAAAAGTCGTAACAAGG	94 °C: 3 min, (94 °C: 15 s, 54 °C: 15 s, 72 °C: 30 s) ×35 cycles, 72 °C: 5 min
	ITS4	TCCTCCGCTTATTGATATGC	
*tef1-α*	EF1-728F	CATCGAGAAGTTCGAGAAGG	94 °C: 3 min, (94 °C: 15 s, 59.5 °C: 15 s, 72 °C: 30 s) ×35 cycles, 72 °C: 5 min
	EF1-986R	TACTTGAAGGAACCCTTACC	
*tub2*	Bt2a	GGTAACCAAATCGGTGCTGCTTTC	94 °C: 3 min, (94 °C: 15 s, 55 °C: 15 s, 72 °C: 30 s) ×35 cycles, 72 °C: 5 min
	Bt2b	ACCCTCAGTGTAGTGACCCTTGGC	

### ﻿Phylogenetic analyses

The newly sequences generated in this study were analyzed with other related sequences obtained from GenBank (Table 2), based on recent publications (Hsu et al. 2024; Li et al. 2024; Wang et al. 2024; Zhao et al. 2024). *Nonappendiculataquercina* (CBS 116061) and *N.quercina* (CBS 270.82) were used as outgroup taxa. Multiple sequences were aligned using MAFFT version 7 (http://mafft.cbrc.jp/alignment/server/index.html) with default settings (Katoh and Standley 2013). To identify *Pestalotiopsis* taxa, single gene phylogenies were inferred for ITS, *tef1-α* and *tub2*, and the sequences of three loci (ITS, *tef1-α* and *tub2*) were concatenated using the “Concatenate Sequence” function in Phylosuite software v1.2.1 (Zhang et al. 2020) to conduct a multi-locus analysis including maximum-likelihood (ML) and Bayesian inference (BI) methods, and the best evolutionary model was selected for each alignment dataset using ModelFinder (Kalyaanamoorthy et al. 2017) and incorporated into the analyses. For the ML analysis, maximum-likelihood phylogenies were inferred using IQ-TREE (Nguyen et al. 2015) under best partitioned models, and tree stability was evaluated with 10,000 ultrafast bootstraps (Minh et al. 2013). The TIM3e+I+G4 model was selected as the most suitable for ITS data partitions, and the TIM2+F+I+G4 model was selected for *tef1-α* and *tub2* data partition. For the BI analysis, Bayesian inference phylogenies were performed using MrBayes 3.2.6 (Ronquist et al. 2012), in which the best nucleotide substitution model for each locus was identified using ModelFinder of Phylosuite, and the best-fit model was GTR+F+I+G4 for ITS, *tef1-α* and *tub2*. Phylogenetic trees were visualized in FigTree v1.4.2 (http://tree.bio.ed.ac.uk/software/figtree, accessed on 12 September 2024), edited and typeset using Adobe Illustrator 2021. The names of the isolates from the present study are marked in red in the trees.

**Table 2.** Taxa used in the phylogenetic analyses and their GenBank accession numbers. New sequences are in bold.

Species	Strain Number	Host/Substrate	Locality	GenBank Accession Number		
				ITS	*tef1-α*	*tub2*
*Pestalotiopsisabietis*	CFCC 53011 ^T^	*Abiesfargesii*	China	MK397013	MK622277	MK622280
*P.abietis*	CFCC 53012	*Abiesfargesii*	China	MK397014	MK622278	MK622281
*P.adusta*	ICMP 6088 ^T^	Refrigerator door	Fiji	JX399006	JX399070	JX399037
*P.adusta*	MFLUCC 10–146	*Syzygium* sp.	Thailand	JX399007	JX399071	JX399038
*P.aggestorum*	LC 6301 ^T^	*Camelliasinensis*	China	KX895015	KX895234	KX895348
*P.aggestorum*	LC 8186	*Camelliasinensis*	China	KY464140	KY464150	KY464160
*P.alloschemones*	CGMCC 3.23480 ^T^	*Alloschemoneoccidentalis*	China	OR247981	OR361456	OR381056
*P.alloschemones*	LC15841	*Alloschemoneoccidentalis*	China	OR247982	OR361457	OR381057
***P.alpinicola***	**HJAUP C1644.221** ^T^	***Alpiniazerumbet***	**China**	**PP962274**	**PP952249**	**PP952219**
***P.alpinicola***	**HJAUP C1644.222**	***Alpiniazerumbet***	**China**	**PP962275**	**PP952248**	**PP952220**
*P.anacardiacearum*	IFRDCC 2397 ^T^	*Mangiferaindica*	China	KC247154	KC247156	KC247155
*P.anhuiensis*	CFCC 54791 ^T^	*Cyclobalanopsisglauca*	China	ON007028	ON005045	ON005056
*P.aporosae-dioicae*	SAUCC224004 ^T^	*Aporosadioica*	China	OR733506	OR912988	OR912985
*P.aporosae-dioicae*	SAUCC224005	*Aporosadioica*	China	OR733505	OR912989	OR912986
*P.appendiculata*	CGMCC 3.23550 ^T^	*Rhododendrondecorum*	China	OP082431	OP185509	OP185516
*P.arceuthobii*	CBS 434.65 ^T^	*Arceuthobiumcampylopodum*	USA	KM199341	KM199516	KM199427
*P.arengae*	CBS 331.92 ^T^	*Arengaundulatifolia*	Singapore	KM199340	KM199515	KM199426
*P.australasiae*	CBS 114126 ^T^	*Knightia* sp.	New Zealand	KM199297	KM199499	KM199409
*P.australasiae*	CBS 114141	*Protea* sp.	New South Wales	KM199298	KM199501	KM199410
*P.australis*	CBS 111503	*Protea neriifolia × susannae* cv. ‘Pink Ice’	South Africa	KM199331	KM199557	KM199382
*P.australis*	CBS 114193 ^T^	*Grevillea* sp.	New South Wales	KM199332	KM199475	KM199383
*P.biappendiculata*	CGMCC 3.23487 ^T^	*Rhododendron* sp.	China	OR247984	OR361459	OR381059
*P.biappendiculata*	LC4282	*Rhododendron* sp.	China	OR247990	OR361465	OR381065
*P.biappendiculata*	LC4283	*Rhododendron* sp.	China	OR247991	OR361466	OR381066
*P.biciliata*	CBS 124463 ^T^	*Platanus×hispanica*	Slovakia	KM199308	KM199505	KM199399
*P.biciliata*	CBS 236.38	*Paeonia* sp.	Italy	KM199309	KM199506	KM199401
*P.brachiata*	LC 2988 ^T^	*Camellia* sp.	China	KX894933	KX895150	KX895265
*P.brachiata*	LC 8188	*Camellia* sp.	China	KY464142	KY464152	KY464162
*P.brachiata*	LC 8189	*Camellia* sp.	China	KY464143	KY464153	KY464163
*P.brassicae*	CBS 170.26 ^T^	*Brassicanapus*	New Zealand	KM199379	KM199558	–
***P.camelliicola***	**HJAUP C1804.221** ^T^	***Camelliajaponica***	**China**	**PP962357**	**PP952236**	**PP952229**
***P.camelliicola***	**HJAUP C1804.222**	***Camelliajaponica***	**China**	**PP962358**	**PP952235**	**PP952230**
*P.camelliae*	MFLUCC 12–0277 ^T^	*Camelliajaponica*	China	JX399010	JX399074	JX399041
*P.camelliae–oleiferae*	CSUFTCC 08 ^T^	*Camelliaeoleiferae*	China	OK493593	OK507963	OK562368
*P.camelliae–oleiferae*	CSUFTCC 09	*Camelliaeoleiferae*	China	OK493594	OK507964	OK562369
*P.cangshanensis*	CGMCC 3.23544 ^T^	*Rhododendrondelavayi*	China	OP082426	OP185510	OP185517
*P.castanopsidis*	CFCC 54430 ^T^	*Castanopsislamontii*	China	OK339732	OK358493	OK358508
*P.castanopsidis*	CFCC 54305	*Castanopsishystrix*	China	OK339733	OK358494	OK358509
*P.castanopsidis*	CFCC 54384	*Castanopsishystrix*	China	OK339734	OK358495	OK358510
*P.chamaeropis*	CBS 186.71 ^T^	*Chamaeropshumilis*	Italy	KM199326	KM199473	KM199391
*P.chamaeropis*	CFCC 55122	*Quercusaliena*	China	OM746229	OM840001	OM839902
*P.chamaeropis*	CFCC 55023	*Castanopsisfissa*	China	OM746233	OM840005	OM839906
*P.changjiangensis*	CFCC 54314 ^T^	*Castanopsistonkinensis*	China	OK339739	OK358500	OK358515
*P.changjiangensis*	CFCC 54433	*Castanopsishainanensis*	China	OK339740	OK358501	OK358516
*P.changjiangensis*	CFCC 52803	*Cyclobalanopsisaustrocochinchinensis*	China	OK339741	OK358502	OK358517
*P.chaoyangensis*	CFCC 55549 ^T^	*Euonymusjaponicus*	China	OQ344763	OQ410582	OQ410584
*P.chaoyangensis*	CFCC 58805	*Euonymusjaponicus*	China	OQ344764	OQ410583	OQ410585
*P.chiangmaiensis*	MFLUCC 22–0127	*Phyllostachysedulis*	Thailand	OP497990	OP753374	OP752137
*P.chiaroscuro*	BRIP 72970 ^T^	*Sporobolusnatalensis*	Australia	OK422510	OK423753	OK423752
*P.chinensis*	MFLUCC 12–0273 ^T^	NA	China	JX398995	–	–
*P.clavata*	MFLUCC 12–0268 ^T^	*Buxus* sp.	China	JX398990	JX399056	JX399025
*P.colombiensis*	CBS 118553 ^T^	*Eucalyptusurograndis*	Colombia	KM199307	KM199488	KM199421
*P.cratoxyli*	CGMCC 3.23512 ^T^	*Cratoxylumcochinchinense*	China	OR248005	OR361480	OR381080
*P.cratoxyli*	LC8772	*Cratoxylumcochinchinense*	China	OR248004	OR361479	OR381079
*P.cyclobalanopsidis*	CFCC 54328 ^T^	*Cyclobalanopsisglauca*	China	OK339735	OK358496	OK358511
*P.cyclobalanopsidis*	CFCC 55891	*Cyclobalanopsisglauca*	China	OK339736	OK358497	OK358512
***P.cyclosora***	**HJAUP C1724.221** ^T^	***Cyclosorusinterruptus***	**China**	**PP962279**	**PP952247**	**PP952221**
***P.cyclosora***	**HJAUP C1724.222**	***Cyclosorusinterruptus***	**China**	**PP962280**	**PP952246**	**PP952222**
***P.cyclosora***	**HJAUP C1725.221**	***Microlepiamarginata***	**China**	**PP962281**	**PP952245**	**PP952223**
***P.cyclosora***	**HJAUP C1725.222**	***Microlepiamarginata***	**China**	**PP962282**	**PP952244**	**PP952233**
***P.cyclosora***	**HJAUP C1726.221**	***Punicagranatum***	**China**	**PP962283**	**PP952243**	**PP952224**
***P.cyclosora***	**HJAUP C1726.222**	***Punicagranatum***	**China**	**PP962284**	**PP952242**	**PP952232**
*P.daliensis*	CGMCC 3.23548 ^T^	*Rhododendrondecorum*	China	OP082429	OP185511	OP185518
*P.dianellae*	CBS 143421 ^T^	*Dianella* sp.	Australia	MG386051	–	MG386164
*P.digitalis*	MFLU 14–0208 ^T^	*Digitalispurpurea*	New Zealand	KP781879	–	KP781883
*P.dilucida*	LC3232 ^T^	*Camelliasinensis*	China	KX894961	KX895178	KX895293
*P.dilucida*	LC8184	*Camelliasinensis*	China	KY464138	KY464148	KY464158
*P.diploclisiae*	CBS 115449	*Psychotriatutcheri*	China	KM199314	KM199485	KM199416
*P.diploclisiae*	CBS 115587 ^T^	*Diploclisiaglaucescens*	China	KM199320	KM199486	KM199419
*P.disseminata*	CBS 143904	*Perseaamericana*	New Zealand	MH554152	MH554587	MH554825
*P.disseminata*	MEAN 1165	*Pinuspinea*	Portugal	MT374687	MT374699	MT374712
*P.diversiseta*	MFLUCC 12–0287 ^T^	*Rhododendron* sp.	China	JX399009	JX399073	JX399040
*P.doitungensis*	MFLUCC 14–0115	*Dendrobium* sp.	Thailand	MK993574	MK975832	MK975837
*P.dracaenae*	HGUP 4037 ^T^	*Dracaenafragrans*	China	–	MT598644	MT598645
*P.dracaenicola*	MFLUCC 18–0913 ^T^	*Dracaena* sp.	Thailand	MN962731	MN962732	MN962733
*P.dracaenicola*	MFLUCC 18–0914	*Dracaena* sp.	Thailand	MN962734	MN962735	MN962736
*P.dracontomelonis*	MFLU 14–0207	*Dracontomelondao*	Thailand	KP781877	KP781880	–
*P. eleuthero–cocci*	HMJAU 60189	*Eleutherococcusbrachypus*	China	OL996126	–	–
*P. eleuthero–cocci*	HMJAU 60190	*Eleutherococcusbrachypus*	China	OL996127	–	OL898722
*P.endophytica*	MFLUCC 18–0932 ^T^	*Magnoliagarrettii*	Thailand	MW263946	MW417119	–
*P.endophytica*	MFLUCC 18–0946	*Magnoliagarrettii*	Thailand	MW263947	MW729384	–
*P.ericacearum*	IFRDCC 2439 ^T^	*Rhododendrondelavayi*	China	KC537807	KC537814	KC537821
***P.eriobotryae***	**HJAUP C1742.221** ^T^	***Eriobotryajaponica***	**China**	**PP962289**	**PP952238**	**PP952227**
***P.eriobotryae***	**HJAUP C1742.222**	***Eriobotryajaponica***	**China**	**PP962291**	**PP952237**	**PP952228**
*P.etonensis*	BRIP 66615 ^T^	*Sporobolusjacquemontii*	Australia	MK966339	MK977635	MK977634
*P.exudata*	CGMCC 3.23488 ^T^	*Aucubajaponica*	China	OR247985	OR361460	OR381060
*P.exudata*	LC15850	*Aucubajaponica*	China	OR247986	OR361461	OR381061
*P.ficicrescens*	HGUP 861 ^T^	*Camelliajaponica*	China	MZ477311	MZ868328	MZ868301
*P.foliicola*	CFCC 54440 ^T^	*Castanopsisfaberi*	China	ON007029	ON005046	ON005057
*P.foliicola*	CFCC 57359	*Castanopsisfaberi*	China	ON007030	ON005047	ON005058
*P.foliicola*	CFCC 57360	*Castanopsisfaberi*	China	ON007031	ON005048	ON005059
*P.formosana*	NTUCC 17–009 ^T^	Poaceae sp.	China	MH809381	MH809389	MH809385
*P.formosana*	NTUCC 17–010	Poaceae sp.	China	MH809382	MH809390	MH809386
*P.furcata*	MFLUCC 12–0054 ^T^	*Camelliasinensis*	Thailand	JQ683724	JQ683740	JQ683708
*P.furcata*	LC6691	*Camelliasinensis*	China	KX895030	KX895248	KX895363
*P.fusiformis*	CGMCC 3.23495 ^T^	*Rhododendron* sp.	China	OR247995	OR361470	OR381070
*P.fusiformis*	LC15852	*Rhododendron* sp.	China	OR247996	OR361471	OR381071
*P.fusoidea*	CGMCC 3.23545 ^T^	*Rhododendrondelavayi*	China	OP082427	OP185512	OP185519
*P.ganzhouensis*	CGMCC 3.23489 ^T^	*Cinnamomumcamphora*	China	OR247987	OR361462	OR381062
*P.ganzhouensis*	LC5089	*Cinnamomumcamphora*	China	OR247998	OR361473	OR381073
***P.gardeniae***	**HJAUP C1729.221** ^T^	***Gardeniajasminoides***	**China**	**PP962285**	**PP952241**	**PP952225**
***P.gardeniae***	**HJAUP C1729.222**	***Gardeniajasminoides***	**China**	**PP962286**	**PP952240**	**PP952226**
***P.gardeniae***	**HJAUP C1729.223**	***Gardeniajasminoides***	**China**	**PP962287**	**PP952239**	**PP952231**
*P.gaultheriae*	IFRD 411–014 ^T^	*Gaultheriaforrestii*	China	KC537805	KC537812	KC537819
*P.gibbosa*	NOF 3175 ^T^	*Gaultheriashallon*	Canada	LC311589	LC311591	LC311590
*P.grevilleae*	CBS 114127 ^T^	*Grevillea* sp.	Australia	KM199300	KM199504	KM199407
*P.guangdongensis*	ZHKUCC 22–0016 ^T^	*Arengapinnata*	China	ON180762	ON221520	ON221548
*P.guangdongensis*	ZHKUCC 22–0017	*Arengapinnata*	China	ON180763	ON221521	ON221549
*P.guangdongensis*	ZHKUCC 22–0018	*Arengapinnata*	China	ON180764	ON221522	ON221550
*P.guangxiensis*	CFCC 54308 ^T^	*Quercusgriffithii*	China	OK339737	OK358498	OK358513
*P.guangxiensis*	CFCC 54300	*Quercusgriffithii*	China	OK339738	OK358499	OK358514
*P.guiyangensis*	CFCC 70626	*Eriobotryajaponica*	China	PP784740	PP842629	PP842617
*P.guiyangensis*	CFCC 70630	*Rohdeajaponica*	China	PP784741	PP842630	PP842618
*P.guizhouensis*	CFCC 54803	*Cyclobalanopsisglauca*	China	ON007035	ON005052	ON005063
*P.guizhouensis*	CFCC 57364 ^T^	*Cyclobalanopsisglauca*	China	ON007036	ON005053	ON005064
*P.hawaiiensis*	CBS 114491 ^T^	*Leucospermum* sp.	USA	KM199339	KM199514	KM199428
***P.hederae***	**HJAUP C1638.221** ^T^	***Hederahelix***	**China**	**PP962270**	**PP952252**	**PP952234**
***P.hederae***	**HJAUP C1638.222**	***Hederahelix***	**China**	**PP962271**	–	**PP952216**
*P.hispanica*	CBS 115391 ^T^	*Protea* sp.	Spain	MH553981	MH554399	MH554640
*P.hollandica*	CBS 265.33 ^T^	*Sciadopitysverticillata*	Netherlands	KM199328	KM199481	KM199388
*P.hollandica*	MEAN 1091 ^T^	*Pinuspinea*	Portugal	MT374678	MT374691	MT374703
*P.humicola*	CBS 336.97 ^T^	Soil	Papua New Guinea	KM199317	KM199484	KM199420
*P.hunanensis*	CSUFTCC15 ^T^	*Camelliaoleifera*	China	OK493599	OK507969	OK562374
*P.hunanensis*	CSUFTCC18	*Camelliaoleifera*	China	OK493600	OK507970	OK562375
*P.hydei*	MFLUCC 20–0135 ^T^	*Litseapetiolata*	Thailand	MW266063	MW251113	MW251112
*P.iberica*	CAA 1004 ^T^	*Pinusradiata*	Spain	MW732248	MW759038	MW759035
*P.iberica*	CAA 1006	*Pinusradiata*	Spain	MW732249	MW759039	MW759036
*P.inflexa*	MFLUCC 12–0270 ^T^	Unidentified tree	China	JX399008	JX399072	JX399039
*P.intermedia*	MFLUCC 12–0259 ^T^	Unidentified tree	China	JX398993	JX399059	JX399028
*P.italiana*	MFLU 14–0214 ^T^	*Cupressusglabra*	Italy	KP781878	KP781881	KP781882
*P.jesteri*	MFLUCC12–0279	*Fagraeabodenii*	China	JX399012	JX399076	JX399043
*P.jiangsuensis*	CFCC 59538	*Pinusmassoniana*	China	OR533577	OR539186	OR539191
*P.jiangsuensis*	CFCC 59539	*Pinusmassoniana*	China	OR533578	OR539187	OR539192
*P.jiangsuensis*	CFCC 59542	*Pinusmassoniana*	China	OR533581	OR539190	OR539195
*P.jiangxiensis*	LC4399 ^T^	*Camellia* sp.	China	KX895009	KX895227	KX895341
*P.jiangxiensis*	LC4242	*Eurya* sp.	China	KX895035	KX895213	KX895327
*P.jinchanghensis*	LC6636 ^T^	*Camelliasinensis*	China	KX895028	KX895247	KX895361
*P.jinchanghensis*	LC8190	*Camelliasinensis*	China	KY464144	KY464154	KY464164
*P.kaki*	KNU–PT–1804 ^T^	*Diospyroskaki*	Korea	LC552953	LC553555	LC552954
*P.kandelicola*	NCYUCC 19–0355 ^T^	*Kandeliacandel*	China	MT560723	MT563102	MT563100
*P.kenyana*	CBS 442.67 ^T^	*Coffea* sp.	Kenya	KM199302	KM199502	KM199395
*P.kenyana*	LC6633	*Camelliasinensis*	China	KX895027	KX895246	KX895360
*P.kenyana*	CFCC 54962	*Quercusaliena*	China	OM746237	OM840009	OM839910
*P.kenyana*	CFCC 54805	*Cyclobalanopsisglauca*	China	OM746253	OM840025	OM839926
*P.kenyana*	CFCC 55088	*Castanopsisfissa*	China	OM746254	OM840026	OM839927
*P.knightiae*	CBS 111963	*Knightia* sp.	New Zealand	KM199311	KM199495	KM199406
*P.knightiae*	CBS 114138 ^T^	*Knightia* sp.	New Zealand	KM199310	KM199497	KM199408
*P.krabiensis*	MFLUCC 16–0260 ^T^	*Pandanus* sp.	Thailand	MH388360	MH388395	MH412722
*P.leucadendri*	CBS 121417 ^T^	*Leucadendron* sp.	South Africa	MH553987	MH554412	MH554654
*P.licualicola*	HGUP 4057 ^T^	*Licualagrandis*	China	KC492509	KC481684	KC481683
*P.lijiangensis*	CFCC 50738 ^T^	Castanopsiscarlesiivar.spinulosa	China	KU860520	KU844185	KU844184
*P.linearis*	MFLUCC 12–0271 ^T^	*Trachelospermum* sp.	China	JX398992	JX399058	JX399027
*P.linguae*	ZHKUCC 22–0159 ^T^	*Pyrrosialingua*	China	OP094104	OP186110	OP186108
*P.linguae*	ZHKUCC 22–0160	*Pyrrosialingua*	China	OP094103	OP186109	OP186107
*P.lithocarpi*	CFCC 55100 ^T^	*Lithocarpuschiungchungensis*	China	OK339742	OK358503	OK358518
*P.lithocarpi*	CFCC 55893	*Lithocarpuschiungchungensis*	China	OK339743	OK358504	OK358519
*P.lobata*	CGMCC 3.23467 ^T^	*Lithocarpusglaber*	China	OR247976	OR361451	OR381051
*P.lobata*	LC15843	*Lithocarpusglaber*	China	OR247977	OR361452	OR381052
*P.loeiana*	MFLUCC 22–0123 ^T^	Dead leaves	Thailand	OP497988	OP737881	OP713769
*P.longiappendiculata*	LC3013	*Camelliasinensis*	China	KX894939	KX895156	KX895271
*P.lushanensis*	LC4344 ^T^	*Camellia* sp.	China	KX895005	KX895223	KX895337
*P.lushanensis*	LC8182	*Camellia* sp.	China	KY464136	KY464146	KY464156
*P.lushanensis*	LC8183	*Camellia* sp.	China	KY464137	KY464147	KY464157
*P.lushanensis*	CFCC 54894	*Quercusserrata*	China	OM746282	OM840054	OM839955
*P.macadamiae*	BRIP 63738b ^T^	*Macadamiaintegrifolia*	Australia	KX186588	KX186621	KX186680
*P.macadamiae*	BRIP 63739b	*Macadamiaintegrifolia*	Australia	KX186587	KX186620	KX186679
*P.macadamiae*	BRIP 637441a	*Macadamiaintegrifolia*	Australia	KX186586	KX186619	KX186678
*P.machili*	CGMCC 3.23511 ^T^	*Machilus* sp.	China	OR248003	OR361478	OR381078
***P***. ***machiliana***	**HJAUP C1790.221** ^T^	***Machiluspauhoi***	**China**	**PP962355**	**PP952253**	**PP952214**
***P***. ***machiliana***	**HJAUP C1790.222**	***Machiluspauhoi***	**China**	**PP962356**	**PP952254**	**PP952215**
***P***. ***machiliana***	**HJAUP C1704.221**	***Rhododendronsimsii***	**China**	**PP962276**	**PP952255**	**PP952211**
***P***. ***machiliana***	**HJAUP C1704.222**	***Rhododendronsimsii***	**China**	**PP962277**	**PP952256**	**PP952212**
***P***. ***machiliana***	**HJAUP C1704.223**	***Rhododendronsimsii***	**China**	**PP962278**	**PP952257**	**PP952213**
*P.malayana*	CBS 102220	*Macarangatriloba*	Malaysia	KM199306	KM199482	KM199411
***P***. ***mangifericola***	**HJAUP C1639.221** ^T^	***Mangiferaindica***	**China**	**PP962272**	**PP952251**	**PP952217**
***P***. ***mangifericola***	**HJAUP C1639.222**	***Mangiferaindica***	**China**	**PP962273**	**PP952250**	**PP952218**
*P.manyueyuanani*	NTUPPMCC 18-165 ^T^	*Ophiocordyceps* sp.	China	OR125060	OR126313	OR126306
*P.manyueyuanani*	NTUPPMCC 22-012	*Ophiocordyceps* sp.	China	OR125061	OR126314	OR126307
*P.menhaiensis*	YN3A1 ^T^	*Camelliasinensis*	China	KU252272	KU252401	KU252488
*P.monochaeta*	CBS 144.97 ^T^	*Quercusrobur*	Netherlands	KM199327	KM199479	KM199386
*P.monochaeta*	CBS 440.83	*Taxusbaccata*	Netherlands	KM199329	KM199480	KM199387
*P.multiappendiculata*	CGMCC 3.23514 ^T^	NA	China	OR248008	OR361483	OR381083
*P.multicolor*	CFCC59981 ^T^	*Taxuschinensis*	China	OQ626676	OQ714341	OQ714336
*P.multicolor*	CFCC59982	*Taxuschinensis*	China	OQ771896	OQ779483	OQ779488
*P.nanjingensis*	CSUFTCC20	*Camelliaoleifera*	China	OK493603	OK507973	OK562378
*P.nanjingensis*	CSUFTCC04	*Camelliaoleifera*	China	OK493604	OK507974	OK562379
*P.nanningensis*	CSUFTCC10 ^T^	*Camelliaoleifera*	China	OK493596	OK507966	OK562371
*P.nanningensis*	CSUFTCC11	*Camelliaoleifera*	China	OK493597	OK507967	OK562372
*P.nannuoensis*	SAUCC232203 ^T^	Unknown host	China	OR733504	OR912991	OR863909
*P.nannuoensis*	SAUCC232204	Unknown host	China	OR733503	OR912992	OR863910
*P.neglecta*	TAP1100 ^T^	*Quercusmyrsinaefolia*	Japan	AB482220	LC311600	LC311599
*P.neolitseae*	NTUCC 17–011 ^T^	*Neolitseavillosa*	Taiwan	MH809383	MH809391	MH809387
*P.neolitseae*	CFCC 54590	*Lithocarpusamygdalifolius*	China	OK339744	OK358505	OK358520
*P.novae-hollandiae*	CBS 130973 ^T^	*Banksiagrandis*	Australia	KM199337	KM199511	KM199425
*P.oryzae*	CBS 111522	*Telopea* sp.	USA	KM199294	KM199493	KM199394
*P.oryzae*	CBS 171.26	NA	Italy	KM199304	KM199494	KM199397
*P.oryzae*	CBS 353.69 ^T^	*Oryzasativa*	Denmark	KM199299	KM199496	KM199398
*P.pallidotheae*	MAFF 240993 ^T^	*Pierisjaponica*	Japan	AB482220	LC311585	LC311584
*P.pandanicola*	MFLUCC 16–0255 ^T^	*Pandanus* sp.	Thailand	MH388361	MH388396	MH412723
*P.papuana*	CBS 331.96 ^T^	Coastal soil	Papua New Guinea	KM199321	KM199491	KM199413
*P.papuana*	CBS 887.96	*Cocosnucifera*	Papua New Guinea	KM199318	KM199492	KM199415
*P.parva*	CBS 265.37	*Delonixregia*	NA	KM199312	KM199508	KM199404
*P.parva*	CBS 278.35 ^T^	*Leucothoefontanesiana*	NA	KM199313	KM199509	KM199405
*P.photinicola*	GZCC 16–0028 ^T^	*Photiniaserrulata*	China	KY092404	KY047662	KY047663
*P.phyllostachydis*	ZHKUCC 23–0873 ^T^	NA	China	OR343210	OR367675	OR367676
*P.pini*	MEAN 1092 ^T^	*Pinuspinea*	Portugal	MT374680	MT374693	MT374705
*P.pinicola*	KUMCC 19–0183 ^T^	*Pinusarmandii*	China	MN412636	MN417509	MN417507
*P.piraubensis*	COAD 2165 ^T^	*Psidiumguajava*	Brazil	MH627381	MH643774	MH643773
*P.portugalica*	CBS 393.48 ^T^	NA	Portugal	KM199335	KM199510	KM199422
*P.pruni*	CGMCC 3.23507 ^T^	*Prunuscerasoides*	China	OR248001	OR361476	OR381076
*P.pruni*	LC15860	*Prunuscerasoides*	China	OR248002	OR361477	OR381077
*P.rhaphiolepis*	SAUCC367701 ^T^	*Rhaphiolepisindica*	China	OR733502	OR912994	OR863906
*P.rhaphiolepis*	SAUCC367702	*Rhaphiolepisindica*	China	OR733501	OR912995	OR863907
*P.rhizophorae*	MFLUCC 17–0416 ^T^	*Rhizophoramucronata*	Thailand	MK764283	MK764327	MK764349
*P.rhizophorae*	MFLUCC 17–0417	*Rhizophoramucronata*	Thailand	MK764284	MK764328	MK764350
*P.rhododendri*	IFRDCC 2399 ^T^	*Rhododendronsinogrande*	China	KC537804	KC537811	KC537818
*P.rhodomyrtus*	CFCC 54733	*Quercusaliena*	China	OM746310	OM840082	OM839983
*P.rhodomyrtus*	CFCC 55052	*Cyclobalanopsisaugustinii*	China	OM746311	OM840083	OM839984
*P.rosarioides*	CGMCC 3.23549 ^T^	*Rhododendrondecorum*	China	OP082430	OP185513	OP185520
*P.rosea*	MFLUCC 12–0258 ^T^	*Pinus* sp.	China	JX399005	JX399069	JX399036
*P.rubrae*	CGMCC 3.23499 ^T^	*Quercusrubra*	China	OR247997	OR361472	OR381072
*P.rubrae*	LC8233	*Plagiogyriaglauca*	China	OR248000	OR361475	OR381075
*P.sabal*	ZHKUCC 22–0027	*Sabalmexicana*	China	ON180765	ON221523	ON221551
*P.sabal*	ZHKUCC 22–0029	*Sabalmexicana*	China	ON180767	ON221525	ON221553
*P.scoparia*	CBS 176.25 ^T^	*Chamaecyparis* sp.	China	KM199330	KM199478	KM199393
*P.sequoiae*	MFLUCC 13–0399 ^T^	*Sequoiasempervirens*	Italy	KX572339	–	–
*P.shaanxiensis*	CFCC 54958 ^T^	*Quercusvariabilis*	China	ON007026	ON005043	ON005054
*P.shaanxiensis*	CFCC 57356	*Quercusvariabilis*	China	ON007027	ON005044	ON005055
*P.shandogensis*	JZB340038 ^T^	*Robiniapseudoacacia*	China	MN625275	MN626740	MN626729
*P.shorea*	MFLUCC 12–0314 ^T^	*Shoreaobtusa*	Thailand	KJ503811	KJ503817	KJ503814
*P.sichuanensis*	SC3A21 ^T^	*Camelliasinensis*	China	KX146689	KX146748	KX146807
*P.silvicola*	CFCC 55296 ^T^	*Cyclobalanopsiskerrii*	China	ON007032	ON005049	ON005060
*P.silvicola*	CFCC 54915	*Cyclobalanopsiskerrii*	China	ON007033	ON005050	ON005061
*P.silvicola*	CFCC 57363	*Cyclobalanopsiskerrii*	China	ON007034	ON005051	ON005062
*P.smilacicola*	MFLUCC 22–0124	*Smilaxchina*	Thailand	OP497989	OP737879	OP762674
*P.smilacicola*	MFLUCC 22–0125 ^T^	*Dioscorea* sp.	Thailand	OP497991	OP753376	OP762673
*P.sonneratiae*	CFCC 57392	*Sonneratiaapetala*	China	ON114182	ON086810	ON086814
*P.sonneratiae*	CFCC 57394 ^T^	*Sonneratiaapetala*	China	ON114184	ON086812	ON086816
*P.sonneratiae*	CFCC 57395	*Sonneratiaapetala*	China	ON114185	ON086813	ON086817
*P.spathulata*	CBS 356.86 ^T^	*Gevuinaavellana*	Chile	KM199338	KM199513	KM199423
*P.spathuliappendiculata*	CBS 144035 ^T^	*Phoenixcanariensis*	Australia	MH554172	MH554607	MH554845
*P.suae*	CGMCC 3.23546 ^T^	*Rhododendrondelavayi*	China	OP082428	OP185514	OP185521
*P.taxicola*	CFCC59976 ^T^	*Taxuschinensis*	China	OQ626673	OQ714338	OQ714333
*P.taxicola*	CFCC59978	*Taxuschinensis*	China	OQ771893	OQ779480	OQ779485
*P.telopeae*	CBS 114137	*Protea* sp.	Australia	KM199301	KM199559	KM199469
*P.telopeae*	CBS 114161 ^T^	*Telopea* sp.	Australia	KM199296	KM199500	KM199403
*P.telopeae*	CBS 113606	*Telopea* sp.	Australia	KM199295	KM199498	KM199402
*P.terricola*	CBS 141.69 ^T^	Soil	Pacific Islands	MH554004	MH554438	MH554680
*P.thailandica*	MFLUCC 17–1616 ^T^	*Rhizophoraapiculata*	Thailand	MK764286	MK764330	MK764352
*P.thailandica*	MFLUCC 17–1617	*Rhizophoraapiculata*	Thailand	MK764285	MK764329	MK764351
*P.trachycarpicola*	OP068 ^T^	*Trachycarpusfortunei*	China	JQ845947	JQ845946	JQ845945
*P.trachycarpicola*	IFRDCC 2403	*Podocarpusmacrophyllus*	China	KC537809	KC537816	KC537823
*P.trachycarpicola*	LC4523	*Camelliasinensis*	China	KX895011	KX895230	KX895344
*P.tumida*	CFCC 55158 ^T^	*Rosachinensis*	China	OK560610	OL814524	OM158174
*P.tumida*	CFCC 55159	*Rosachinensis*	China	OK560613	OL814527	OM158177
*P.tumida*	CGMCC 3.23502	NA	China	OR247999	OR361474	OR381074
*P.unicolor*	MFLUCC 12–0276 ^T^	*Rhododendron* sp.	China	JX398999	–	JX399030
*P.unicolor*	MFLUCC 12–0275	Unidentified tree	China	JX398998	JX399063	JX399029
*P.verruculosa*	MFLUCC 12–0274 ^T^	*Rhododendron* sp.	China	JX398996	JX399061	–
*P.wulichongensis*	CGMCC 3.23469 ^T^	Poaceae	China	OR247978	OR361453	OR381053
*P.wulichongensis*	LC15846	Poaceae	China	OR247979	OR361454	OR381054
*P.yanglingensis*	LC 4553 ^T^	*Camelliasinensis*	China	KX895012	KX895231	KX895345
*P.yanglingensis*	LC 3412	*Camelliasinensis*	China	KX894980	KX895197	KX895312
*P.yunnanensis*	HMAS 96359 ^T^	*Podocarpusmacrophyllus*	China	AY373375	–	–
*Nonappendiculataquercina*	CBS 116061 ^T^	*Quercussuber*	Italy	MH553982	MH554400	MH554641
*N.quercina*	CBS 270.82	*Quercuspubescens*	Italy	MH554025	MH554459	MH554701

^T^ = ex–type culture. **BRIP** = Queensland Plant Pathology Herbarium, Brisbane, Australia; **CAA** = Culture collection of Artur Alves, housed at Department of Biology, University of Aveiro, Aveiro, Portugal; **CBS** = culture collection of the Westerdijk Fungal Biodiversity Institute, Utrecht, The Netherlands; **CFCC** = China Forestry Culture Collection Center, China; **CGMCC** = China General Microbiological Culture Collection Center, Beijing, China; **ICMP** = International Collection of Microorganisms from Plants, Auckland, New Zealand; **CSUFTCC** = Central South University of Forestry and Technology Culture Collection, Hunan, China; **GZCC** = Guizhou Academy of Agricultural Sciences Culture Collection, Guizhou, China; **HGUP** = Plant Pathology Herbarium of Guizhou University, Guizhou, China; **HMAS** = Mycological Herbarium, Institute of Microbiology, Chinese Academy of Sciences, Beijing, China. **HMJAU** = Herbarium of Mycology of Jilin Agricultural University, Jilin, China; **SAUCC** = Shandong Agricultural University Culture Collection, Taian, Shandong, China; **ICMP** = International Collection of Microorganisms from Plants, Auckland, New Zealand; **IFRDCC** = International Fungal Research and Development Culture Collection, Kunming, Yunnan China; **KNU** = Kyungpook National University, Daegu, South Korea; **KUMCC** = Kunming Institute of Botany Culture Collection, Yunnan, China; **LC** = working collection of Lei Cai, housed at the Institute of Microbiology, Chinese Academy of Sciences, Beijing, China; **MAFF** = Ministry of Agriculture, Forestry and Fisheries, Tsukuba, Ibaraki, Japan; **MEAN** = Instituto Nacional de Investigação Agrária e Veterinária I. P.; **MFLU** = Mae Fah Luang University Herbarium, Thailand; **MFLUCC** = Mae Fah Luang University Culture Collection, Chiang Rai, Thailand; **NCYUCC** = The National Chiayi University Culture Collection, Jiayi, Taiwan; **NOF** = The Fungus Culture Collection of the Northern Forestry Centre, Alberta, Canada; **NTUCC** = The Department of Plant Pathology and Microbiology, National Taiwan University Culture Collection, Taipei, Taiwan China; **TAP** = Tamagawa University, Tokyo, Japan; **ZHKUCC** = the culture collection of Zhongkai University of Agriculture and Engineering, Guangzhou City, Guangdong, China; ITS = internal transcribed spacer; *tub2* = β–tubulin; *tef1-α* = translation elongation factor1–α.

## ﻿Results

### ﻿Molecular phylogeny

**Figure 1.**
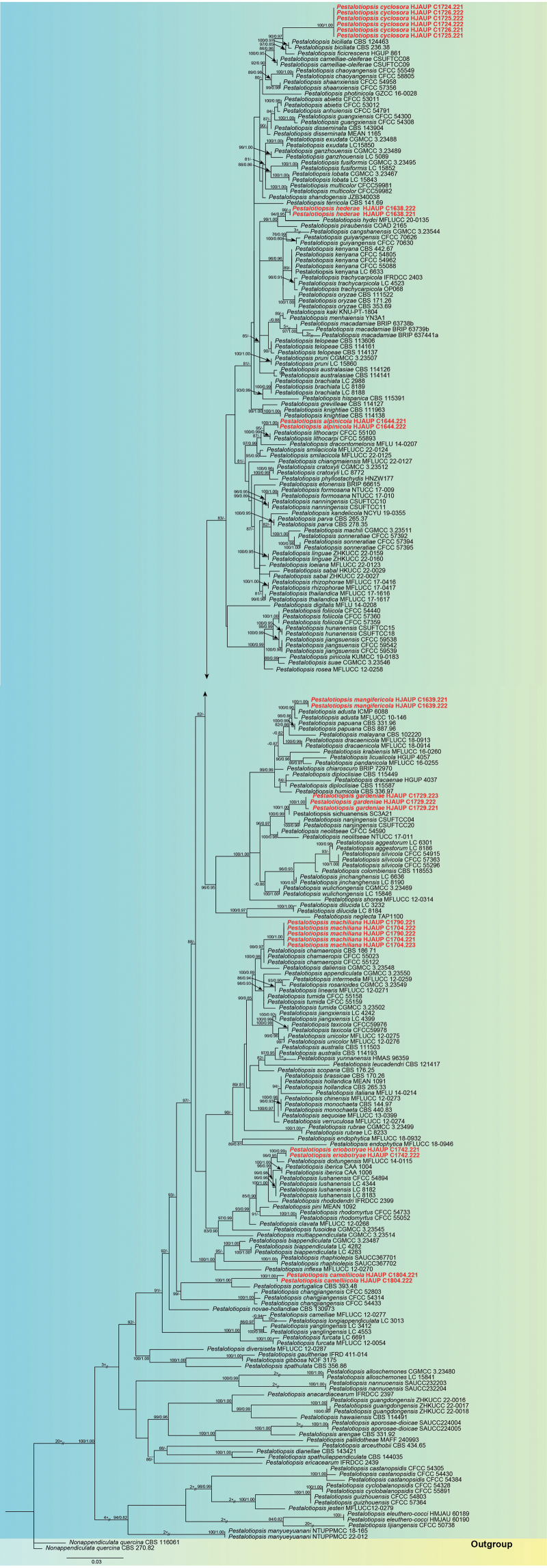
Phylogenetic relationship of *Pestalotiopsis* based on concatenated sequences of ITS, *tef1-α* and *tub2* sequence data. The ML and BI bootstrap support values above 80% and 0.80 are given above the nodes. Bar = 0.03 substitution per nucleotide position. The tree is rooted to *Nonappendiculataquercina* (CBS 116061) and *N.quercina* (CBS 270.82). The strains from the present study are marked in red. Some branches are shortened according to the indicated multipliers to fit the page size, and these are indicated by the symbol (//).

To identify the isolated *Pestalotiopsis* strains, the ITS sequence data were used for initial identification in the present study. By the BLASTn analysis of ITS sequence, 24 strains were categorised into the genus *Pestalotiopsis*. Subsequently, based on maximum-likelihood (ML) and Bayesian inference (BI), the combined analysis of ITS, *tef1-α* and *tub2* gene data was used to construct phylogenetic trees for further determination of the phylogenetic position of these strains. The phylogenetic results represented by the best-scoring ML consensus tree (lnL = –14416.332) are shown in Fig. 1. The 24 isolates obtained from different plants in our study nested within the known *Pestalotiopsis* species with reliable support values. In the multi-loci phylogenies of ITS, *tef1-α* and *tub2*, a total of 266 strains representing 147 accepted species were comprised in the final alignment matrix of *Pestalotiopsis*. *Nonappendiculataquercina* (CBS 116061) and *N.quercina* (CBS 270.82) served as outgroups. The combined data set (ITS: 1–510, *tef1-α*: 511–891 and *tub2*: 892–1284) was composed of 684 distinct patterns, 468 parsimony informative sites, 103 singleton sites, and 713 constant sites. A total of three single-locus data sets, ITS, *tef1-α* and *tub2*, contained 107, 181 and 180 parsimony informative sites, respectively. Combining morphological characteristics and molecular phylogenetic analyses, the 24 strains in this study were introduced as eight new species, namely *Pestalotiopsisalpinicola*, *P.camelliicola*, *P.cyclosora*, *P.eriobotryae*, *P.gardeniae*, *P.hederae*, *P.machiliana* and *P.mangifericola*.

### ﻿Taxonomy

#### ﻿ Pestalotiopsis alpinicola

Taxon classificationFungiAmphisphaerialesSporocadaceae

X.X. Luo & Jian Ma sp. nov.

E2787537-750E-59B0-80C5-6C349542F26F

Index Fungorum: IF902319

Fig. 2

##### Type.

China • Yunnan Province, Xishuangbanna Dai Autonomous Prefecture, Mengla County, Menglun Town, Tropical Botanical Garden, on diseased leaves of *Alpiniazerumbet*, 23 June 2022, X.X. Luo (holotype HJAUP M1644.221; ex-type living culture HJAUP C1644.221).

**Figure 2.**
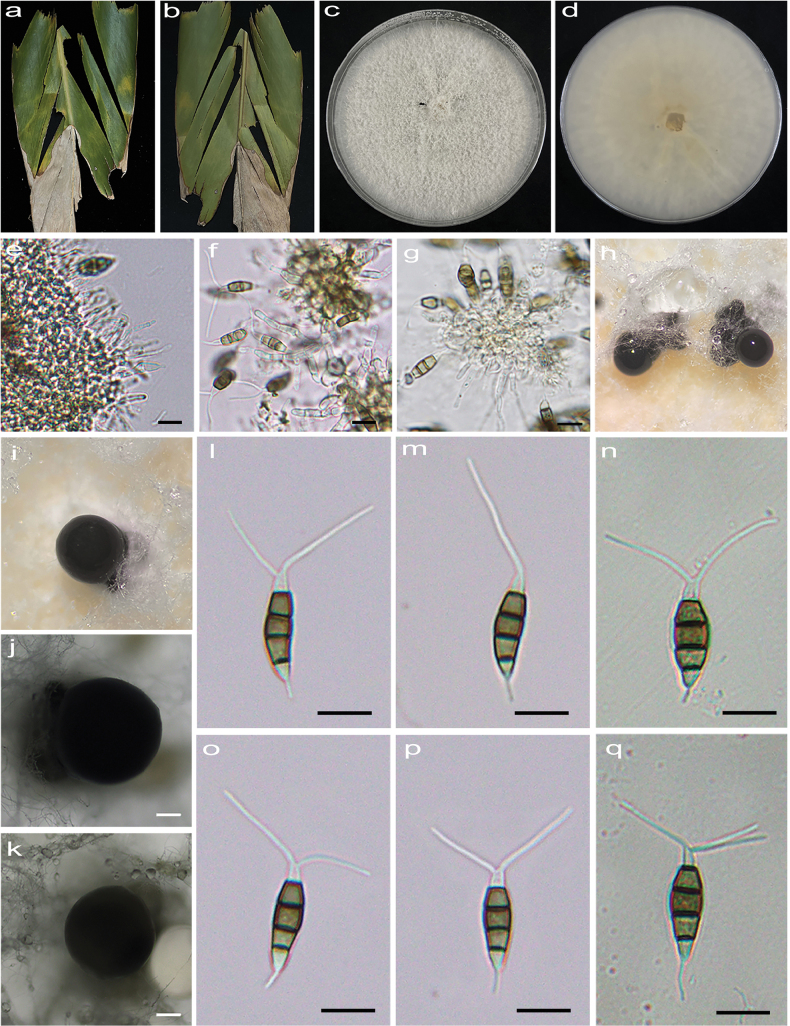
*Pestalotiopsisalpinicola* (HJAUP C1644.221, ex-type) **a, b** leaf of host plant (front and reverse) **c, d** culture on PDA (front and reverse) **e–g** conidiogenous cells and conidia **h–k** conidiomata **l–q** conidia. Scale bars: 200 µm (**j, k**); 10 µm (**e–g, l–q**).

##### Etymology.

Referring to the host genus, *Alpinia* from which it was collected.

##### Description.

Leaf tip blight and irregular pallid leaf spots. Asexual morph on PDA: Conidiomata acervular, globose, 710–1110 μm diam., solitary or aggregated in clusters, black. Conidiophores indistinct and reduced to conidiogenous cells. Conidiogenous cells hyaline, smooth, cylindrical to ampulliform. Conidia fusiform, straight or slightly curved, 18.1–21.8 × 4.7–5.9 μm (x̄ = 19.7 × 5.5 μm, n = 50), 4-septate, slightly constricted at the septa; basal cell conical, 2.6–4.4 μm (x̄ = 3.6 μm) long, hyaline or sometimes pale brown, smooth, thin-walled, with a single filiform appendage, unbranched, 3.6–6.2 μm (x̄ = 5.1 μm) long; three median cells doliiform to cylindrical, smooth, 10–13 μm (x̄ = 12 μm) long, concolorous or sometimes darker at the two upper cells, somewhat constricted at the septa, second cell from the base pale brown to brown, 3.5–4.5 µm (x̄ = 4.1 μm) long, third cell brown, 3.3–4.2 µm (x̄ = 3.8 μm) long, fourth cell pale brown to brown, 3.6–4.5 µm (x̄ = 4.1 μm) long; apical cell conical to acute, hyaline, smooth, thin-walled, 3.1–4.5 µm (x̄ = 3.6 μm) long, with 1–3 (mostly 2) filiform appendages, arising from the apical crest, unbranched, 13.1–20.9 µm long. Sexual morph not observed.

##### Culture characteristics.

Colonies on PDA grow fast, flat and spreading, growing all over the Petri dish after 2 weeks at 25 °C in darkness, white, with flocculent aerial mycelium and entire edge, forming black conidiomata, and reverse pale straw.

##### Additional specimen examined.

China • Yunnan Province, Xishuangbanna Dai Autonomous Prefecture, Mengla County, Menglun Town, Tropical Botanical Garden, 23 June 2022, X.X. Luo. On diseased leaves of *Alpiniazerumbet*; paratype HJAUP M1644.222, living culture HJAUP C1644.222.

##### Note.

Two strains (HJAUP C1644.221 and HJAUP C1644.222) of *Pestalotiopsisalpinicola* isolated from leaf spots of *Alpiniazerumbet* clustered with *P.lithocarpi* (CFCC 55100 and CFCC 55893) with 95% ML/0.68 BI bootstrap support (Fig. 1). The ex-type strain HJAUP C1644.221 is closely related to *P.lithocarpi* (CFCC 55100) and comparisons of their nucleotides showed 20 bp differences (2%, including zero gap) nucleotide differences in three loci. Moreover, *P.alpinicola* is morphologically distinguished from *P.lithocarpi* Ning Jiang by its smaller conidia (4.7–5.9 μm vs. 6–7 μm) with shorter three median cells (10–13 μm vs. 12.5–14 μm) and fewer apical appendages (1–2 vs. 3–4) (Jiang et al. 2022).

#### ﻿ Pestalotiopsis camelliicola

Taxon classificationFungiAmphisphaerialesSporocadaceae

X.X. Luo & Jian Ma sp. nov.

8F7F9CD3-D712-5575-9AD9-FB14C7CF8DEF

Index Fungorum: IF902320

Fig. 3

##### Type.

China • Jiangxi Province, Jingdezhen City, Changjiang District, Jingdezhen Botanical Garden, on diseased leaves of *Camelliajaponica*, 3 November 2022, X.X. Luo (holotype HJAUP M1804.221; ex-type living culture HJAUP C1804.221).

**Figure 3.**
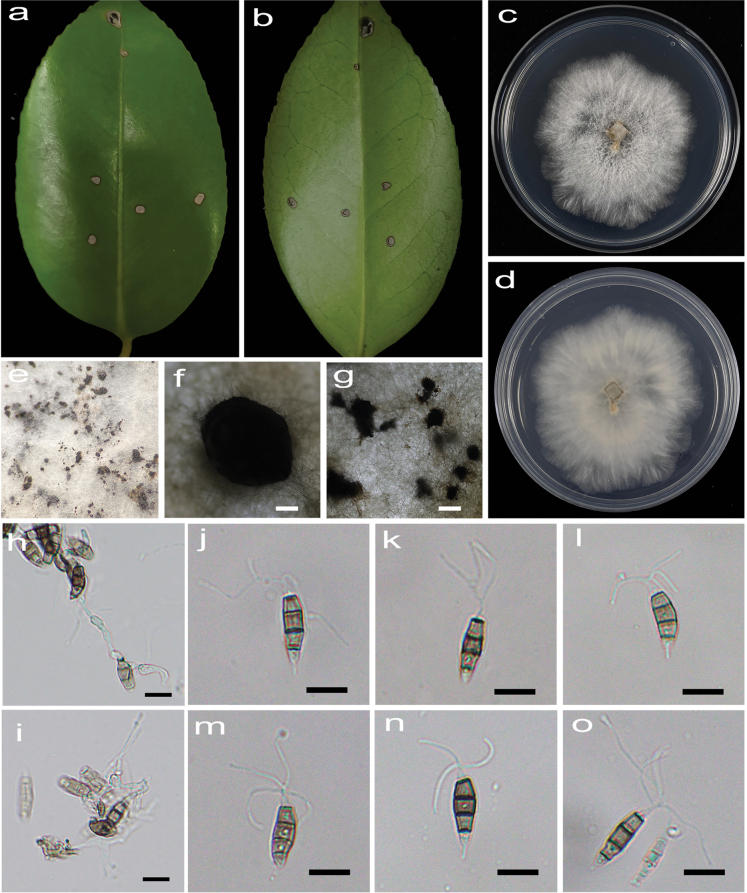
*Pestalotiopsiscamelliicola* (HJAUP C1804.221, ex-type) **a, b** leaf of host plant (front and reverse) **c, d** culture on PDA (front and reverse) **e–g** conidiomata **h, i** conidiogenous cells and conidia **j–o** conidia. Scale bars: 200 µm (**f, g**); 10 µm (**h–o**).

##### Etymology.

Referring to the host genus from which it was collected, *Camelliajaponica*.

##### Description.

Regular leaf spots, grey white in the center, and brown to dark brown at the margin. Asexual morph on PDA: Conidiomata acervular, 470–1320 μm diam., superficial, solitary or aggregated in clusters, dark brown. Conidiophores indistinct and reduced to conidiogenous cells. Conidiogenous cells hyaline, smooth, cylindrical to ampulliform. Conidia fusiform, straight or slightly curved, 14.9–22.2 × 5.4–7.6 μm (x̄ = 18.1 × 6.3 μm, n = 50), 4-septate, mostly with one minute guttules in each cell, slightly constricted at the septa; basal cell conical, 1.8–4 μm (x̄ = 2.8 μm), pale brown, smooth, thin-walled, with a single filiform appendage, unbranched, 1.7–5.2 μm (x̄ = 2.9 μm) long; three median cells doliiform to cylindrical, smooth, 11–14.4 μm (x̄ = 12.4 μm), concolorous, pale brown to brown, somewhat constricted at the septa, second cell from the base 3.8–5.3 µm (x̄ = 4.3 μm) long, third cell 3.6–4.7 µm (x̄ = 4.2 μm) long, fourth cell 3.2–5 µm (x̄ = 4 μm) long); apical cell conical to acute, hyaline, smooth, thin-walled, 2.2–3.8 µm (x̄ = 2.9 μm) long, with 2–4 (mostly 3) filiform appendages, arising from the apical crest, branched, 9.5–20.3 µm (x̄ = 12.4 μm) long. Sexual morph: not observed.

##### Culture characteristics.

Colonies on PDA grow fast, filamentous, reaching 56–62 mm diam. after 5 days at 25 °C in darkness, white, with flocculent mycelium and entire edge, forming black, brown conidiomata, and reverse pale orange.

##### Additional specimen examined.

China • Jiangxi Province, Jingdezhen City, Changjiang District, Jingdezhen Botanical Garden, 3 November 2022, X.X. Luo. On diseased leaves of *Camelliajaponica*, paratype HJAUP M1804.222, living culture HJAUP C1804.222.

##### Note.

Two strains (HJAUP C1804.221 and HJAUP C1804.222) of *Pestalotiopsiscamelliicola* isolated from leaf spots of *Camelliajaponica* formed a distinct clade sister to *P.portugalica* (CBS 393.48) with 100% ML/1.00 BI bootstrap support (Fig. 1). The ex-type strain HJAUP C1804.221 is closely related to *P.portugalica* (CBS 393.48) and comparisons of their nucleotides showed 20 bp differences (2%, including four gaps) nucleotide differences in three loci. Moreover, *P.camelliicola* is morphologically distinguished from *P.portugalica* Maharachch., K.D. Hyde & Crous in its solitary or scattered conidiomata and conidia with more apical filiform appendages (2–4 vs. 1–3). In addition, the conidia of *P.camelliicola* usually have one minute guttule at each cell, which are not observed in *P.portugalica* (Maharachchikumbura et al. 2014).

#### ﻿ Pestalotiopsis cyclosora

Taxon classificationFungiAmphisphaerialesSporocadaceae

X.X. Luo & Jian Ma sp. nov.

1624DBB5-CE01-5760-8CB0-1D8F7F263595

Index Fungorum: IF902321

Fig. 4

##### Type.

China • Jiangxi Province, Xinyu City, Yushui District, Baoshi Park, on diseased leaves of *Cyclosorusinterruptus*, 2 November 2022, X.X. Luo (holotype HJAUP M1724.221; ex-type living culture HJAUP C1724.221).

**Figure 4.**
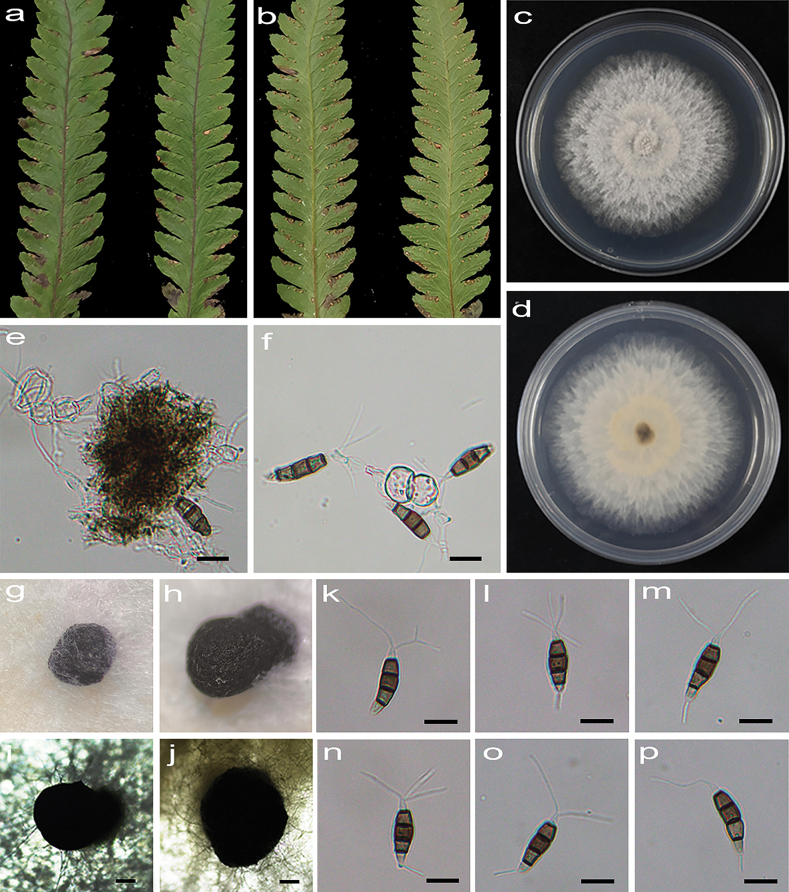
*Pestalotiopsiscyclosora* (HJAUP C1724.221) **a, b** leaf of host plant (front and reverse) **c, d** culture on PDA (front and reverse) **e, f** conidiogenous cells and conidia **g–j** conidiomata **k–p** conidia. Scale bars: 200 µm (**i, j**); 10 µm (**e, f, k–p**).

##### Etymology.

Referring to the host genus, *Cyclosorus* from which it was collected.

##### Description.

Regular leaf spots, yellowish to grey white in the center, and dark brown at the margin. Asexual morph on PDA: Conidiomata acervular, globose, 460–780 μm diam., solitary, black. Conidiophores indistinct and reduced to conidiogenous cells. Conidiogenous cells hyaline, smooth, cylindrical to ampulliform. Conidia fusiform, straight or slightly curved, 16.3−26.1 × 5.4–7.1 μm (x̄ = 21.3 × 6.4 μm, n = 50), 4-septate, slightly constricted at the septa; basal cell conical, 2.7–4.7 μm (x̄ = 3.5 μm), hyaline or sometimes pale brown, smooth, thin-walled, with a single filiform appendage, unbranched, 4.1–10.6 μm (x̄ = 7.7 μm) long; three median cells doliiform to cylindrical, smooth, 11–17.1 μm (x̄ = 14.1 μm), concolorous or sometimes darker at the two upper cells, somewhat constricted at the septa, second cell from the base brown, 3.9–6.2 µm (x̄ = 4.8 μm) long, third cell brown to dark brown, 3.9−5.6 µm (x̄ = 4.7 μm) long, fourth cell brown, 3.8–5.7 µm (x̄ = 4.8 μm) long); apical cell conical to acute, hyaline, smooth, thin-walled, 2.6–4.2 µm (x̄ = 3.6 μm) long, with 1–4 (mostly 2 or 3) filiform appendages, arising from the apical crest, sometimes branched, 12.5–29.8 µm (x̄ = 20.1 μm) long. Sexual morph not observed.

##### Culture characteristics.

Colonies on PDA grow fast, filamentous to circular, reaching 62–69 cm diam. after 5 days at 25 °C in darkness, regular edge, white, with filamentous aerial mycelium and entire edge, and reverse pale orange.

##### Additional specimen examined.

China • Jiangxi Province, Xinyu City, Yushui District, Baoshi Park, 2 November 2022, X.X. Luo. On diseased leaves of *Cyclosorusinterruptus*, paratype HJAUP M1724.222, living culture HJAUP C1724.222; on diseased leaves of *Microlepiamarginata*, paratype HJAUP M1725.221, living culture HJAUP C1725.221; on diseased leaves of *Microlepiamarginata*, paratype HJAUP M1725.222, living culture HJAUP C1725.222 • Yingtan City, Guixi County, Shangqing Town, Longhu Mountain National Forest Park, 3 November 2022, X.X. Luo. On diseased leaves of *Punicagranatum*, paratype HJAUP M1726.221, living culture HJAUP C1726.221; on diseased leaves of *Punicagranatum*, paratype HJAUP M1726.222, living culture HJAUP C1726.222.

##### Notes.

Six strains (HJAUP C1724.221, HJAUP C1724.222, HJAUP C1725.221, HJAUP C1725.222, HJAUP C1726.221 and HJAUP C1726.222) of *Pestalotiopsiscyclosora* isolated from leaf spots of *Cyclosorusinterruptus*, *Microlepiamarginata* and *Punicagranatum* clustered as a sister taxon to the clade containing *P.ficicrescens* (HGUP 861) and *P.biciliata* (CBS 124463 and CBS 236.38) with 90% ML/0.97 BI bootstrap support (Fig. 1). The ex-type strain HJAUP C1724.221 is closely related to *P.ficicrescens* (HGUP 861) and *P.biciliata* (CBS 124463), and comparisons of their nucleotides showed 18 bp differences (2%, including three gaps) and 12 bp differences (1%, including two gaps) nucleotide differences in three loci, respectively. Moreover, *P.cyclosora* is morphologically distinguished from *P.ficicrescens* Qi Yang & Yong Wang bis in its conidia with darker median cells and longer filiform appendages at both ends (apical appendages: 12.5–29.8 µm vs. 10.5–18 µm, basal appendage: 4.1–10.6 μm vs. 3.5–7 µm), and more apical appendages (1–4 vs. 2–3) in apical cell (Hyde et al. 2023). *Pestalotiopsiscyclosora* is also different from *P.biciliata* Maharachch., K.D. Hyde & Crous, which has verruculose conidia with concolourous, olivaceous median cells and longer basal cell (4–7 μm vs. 2.7–4.7 μm) bearing two appendages (Maharachchikumbura et al. 2014).

#### ﻿ Pestalotiopsis eriobotryae

Taxon classificationFungiAmphisphaerialesSporocadaceae

X.X. Luo & Jian Ma sp. nov.

AE6D335E-2858-53FD-B38D-502CF5C4C292

Index Fungorum: IF902322

Fig. 5

##### Type.

China • Jiangxi Province, Yingtan City, Guixi County, Shangqing Town, Longhu Mountain National Forest Park, on diseased leaves of *Eriobotryajaponica*, 3 November 2022, X.X. Luo (holotype HJAUP M1742.221; ex-type living culture HJAUP C1742.221).

**Figure 5.**
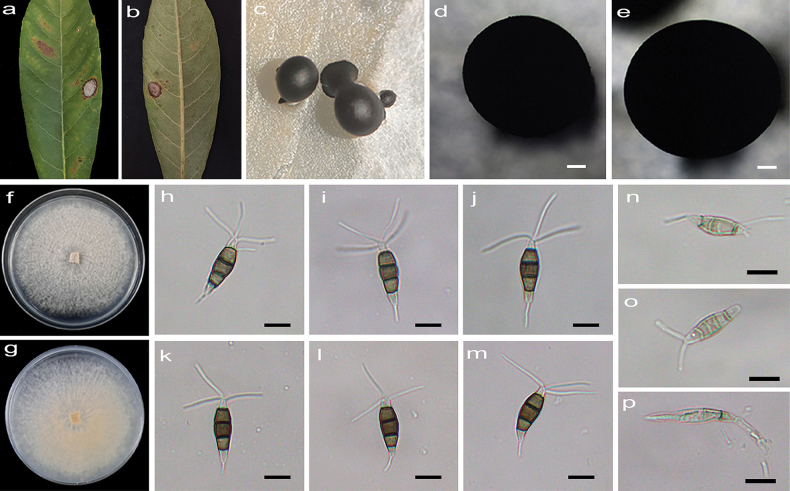
*Pestalotiopsiseriobotryae* (HJAUP C1742.221, ex-type) **a, b** leaf of host plant (front and reverse) **c–e** conidiomata **f, g** culture on PDA (front and reverse) **h–m** conidia **n–p** immature conidia. Scale bars: 200 µm (**d, e**); 10 µm (**h–p**).

##### Etymology.

Referring to the host genus, *Eriobotrya* from which it was collected.

##### Description.

Regular leaf spots, grey white in the center with black-spotted acervuli, and dark brown at the margin with rusty halo. Asexual morph on PDA: Conidiomata acervular, globose, 839–2203 μm diam., solitary or aggregated in clusters, black. Conidiophores indistinct and reduced to conidiogenous cells. Conidiogenous cells hyaline, smooth, cylindrical to ampulliform. Conidia fusiform, straight or slightly curved, 18.3–29.2 × 6.5–9 μm (x̄ = 23.7 × 7.7 μm, n = 50), 4-septate, slightly constricted at the septa, basal cell conical, 2.8–5.3 μm (x̄ = 4 μm), pale brown to subhyaline, smooth, thin-walled, with a single filiform appendage, unbranched, 4.1–11.5 μm (x̄ = 7.1 μm) long; three median cells doliiform to cylindrical, smooth, 12.1–18.6 μm (x̄ = 15.4 μm), concolorous or sometimes darker at the central cell or the two upper cells, somewhat constricted at the septa, second cell from the base pale brown, 3.4–6.9 µm (x̄ = 5 μm) long, third cell medium to dark brown, 3.7–6.2 µm (x̄ = 5.1 μm) long, fourth cell pale to medium brown, 4.4–6.5 µm (x̄ = 5.4 μm) long; apical cell conical, hyaline, smooth, thin-walled, 3.4–5.3 µm (x̄ = 4.2 μm) long, with 3–4 (mostly 3) filiform appendages, arising from the apex of the apical cell each at a different point, unbranched, 14.5–29.2 µm (x̄ = 18.9 μm) long. Sexual morph not observed.

##### Culture characteristics.

Colonies on PDA grow fast, filamentous to circular, reaching 81–85 mm diam. after 5 days at 25 °C in darkness, white to buff, with flocculent mycelium and entire edge, forming black conidiomata, and reverse pale orange.

##### Additional specimen examined.

China • Jiangxi Province, Yingtan City, Guixi County, Shangqing Town, Longhu Mountain National Forest Park, 3 November 2022, X.X. Luo. On diseased leaves of *Eriobotryajaponica*, paratype HJAUP M1742.222, living culture HJAUP C1742.222.

##### Note.

Two strains (HJAUP C1742.221 and HJAUP C1742.222) of *Pestalotiopsiseriobotryae* isolated from leaf spots of *Eriobotryajaponica* formed a well-supported clade phylogenetically close to *P.doitungensis* (MFLUCC 14–0115) with 99% ML/0.95 BI bootstrap support (Fig. 1). The ex-type strain HJAUP C1742.221 is closely related to *P.doitungensis* (MFLUCC 14–0115) and comparisons of their nucleotides showed 17 bp differences (2%, including three gaps) nucleotide differences in three loci. Moreover, *P.eriobotryae* is morphologically distinguished from *P.doitungensis* X.Y. Ma, K.D. Hyde & J.C. Kang in its wider conidia (6.5–9.0 μm vs. 5.5–6.5 μm) with more and longer apical filiform appendages (3–4 vs. 2–3, 14.5–29.2 µm vs. 4–12 μm) (Ma et al. 2019).

#### ﻿ Pestalotiopsis gardeniae

Taxon classificationFungiAmphisphaerialesSporocadaceae

X.X. Luo & Jian Ma sp. nov.

04F18339-8988-5C83-A24A-BF2CF70E3522

Index Fungorum: IF902323

Fig. 6

##### Type.

China • Jiangxi Province, Yingtan City, Guixi County, Shangqing Town, Longhu Mountain National Forest Park, on diseased leaves of *Gardeniajasminoides*, 23 June 2022, X.X. Luo (holotype HJAUP M1729.221; ex-type living culture HJAUP C1729.221).

**Figure 6.**
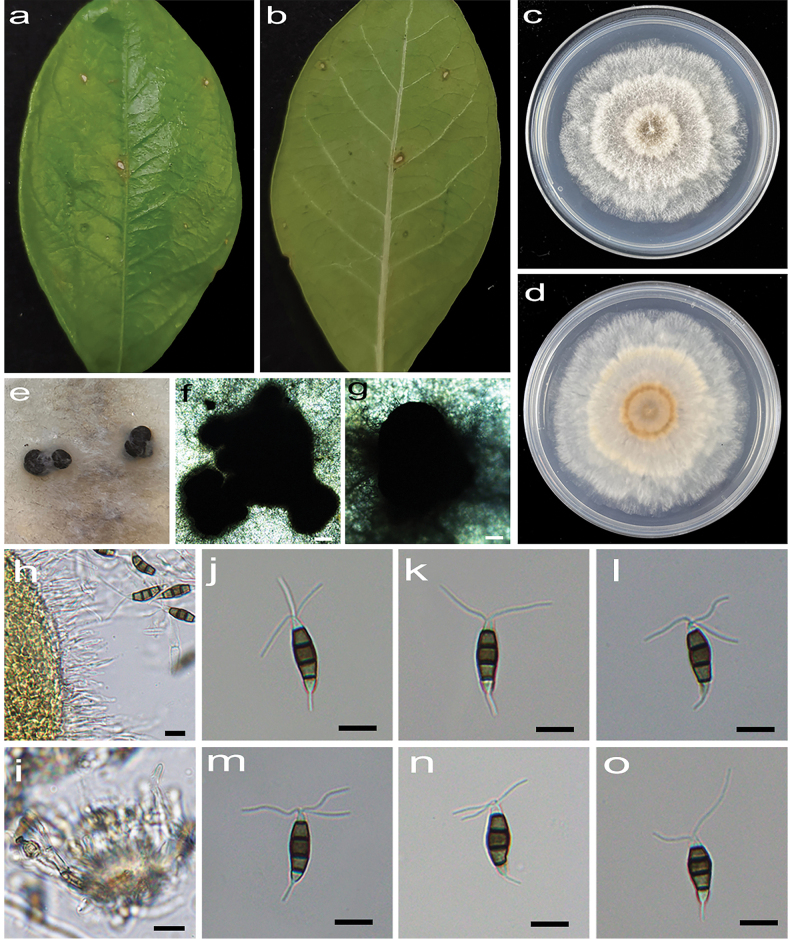
*Pestalotiopsisgardeniae* (HJAUP C1729.221, ex-type) **a, b** leaf of host plant (front and reverse) **c, d** culture on PDA (front and reverse) **e–g** conidiomata **h, i** conidiogenous cells and conidia **j–o** conidia. Scale bars: 200 µm (**f, g**); 10 µm (**h–o**).

##### Description.

Regular leaf spots, grey white in center, and pale brown at margin with yellowish halo. Asexual morph on PDA: Conidiomata acervular, globose or subglobular, 763–955 μm diam., solitary or aggregated, black. Conidiophores indistinct and reduced to conidiogenous cells. Conidiogenous cells hyaline, smooth, cylindrical to ampulliform. Conidia fusiform, straight or slightly curved, 17.4–25.4 × 5.3–6.7 μm (x̄ = 21.9 × 6 μm, n = 50), 4-septate, slightly constricted at the septa; basal cell conical, 3.4–6.4 μm (x̄ = 5.1 μm), pale brown to subhyaline, smooth, thin-walled, with a single filiform appendage, unbranched, 2.9–4.7 μm (x̄ = 3.9 μm) long; three median cells doliiform to cylindrical, 11–14.7 μm (x̄ = 13.2 μm), concolorous or sometimes darker at the central cell or the two upper cells, somewhat constricted at the septa, second cell from the base pale brown, 3.4–5.1 (x̄ = 4.3 μm) µm long, third cell medium to dark brown, 3.7–5.3 µm (x̄ = 4.4 μm) long, fourth cell pale to medium brown, 3.7–5.4 µm (x̄ = 4.5 μm) long; apical cell conical to acute, hyaline, smooth, thin-walled, 2.9–4.3 µm (x̄ = 3.6 μm) long, with 2–3 (mostly 3) filiform appendages, arising from the apical crest, unbranched, 10–20.6 µm (x̄ = 14.4 μm) long. Sexual morph not observed.

##### Culture characteristics.

Colonies on PDA grow fast, filamentous to circular, reaching 70–75 mm diam. after 5 days at 25 °C in darkness, white, with flocculent aerial mycelium and entire edge, forming black conidiomata, and reverse pale orange.

##### Additional specimen examined.

China • Jiangxi Province, Yingtan City, Guixi County, Shangqing Town, Longhu Mountain National Forest Park, 23 June 2022, X.X. Luo. On diseased leaves of *Gardeniajasminoides*, paratype HJAUP M1729.222, living culture HJAUP C1729.222; on diseased leaves of *Gardeniajasminoides*, paratype HJAUP M1729.223, living culture HJAUP C1729.223.

##### Note.

Three strains (HJAUP C1729.221, HJAUP C1729.222 and HJAUP C1729.223) of *Pestalotiopsisgardeniae* isolated from leaf spots of *Gardeniajasminoides* formed a distinct clade sister to *P.sichuanensis* (SA3A21) with 100% ML/1.00 BI bootstrap support (Fig. 1). The ex-type strain HJAUP C1729.221 is closely related to *P.sichuanensis* (SA3A21) and comparisons of their nucleotides showed 3 bp differences (1%, including zero gap) nucleotide differences in three loci. Moreover, *P.gardeniae* is morphologically distinguished from *P.sichuanensis* Y.C. Wang, X.C. Wang & Y.J. Yang in its larger conidia (17.4–25.4 × 5.3–6.7 μm vs. 8.6–12.5 × 2.6–3.7 μm) with longer apical filiform appendages (10–20.6 μm vs. 2.6–9.2 μm) (Wang et al. 2019).

#### ﻿ Pestalotiopsis hederae

Taxon classificationFungiAmphisphaerialesSporocadaceae

X.X. Luo & Jian Ma sp. nov.

EE75BA2A-EF12-5CFC-BBB5-C5F2AC0E4319

Index Fungorum: IF902324

Fig. 7

##### Type.

China • Yunnan Province, Jinghong City, Menghan Town, Xishuangbanna Dai Nationality Garden; on diseased leaves of *Hederahelix*; 22 June 2022, X.X. Luo (holotype HJAUP M1638.221; ex-type living culture HJAUP C1638.221).

**Figure 7.**
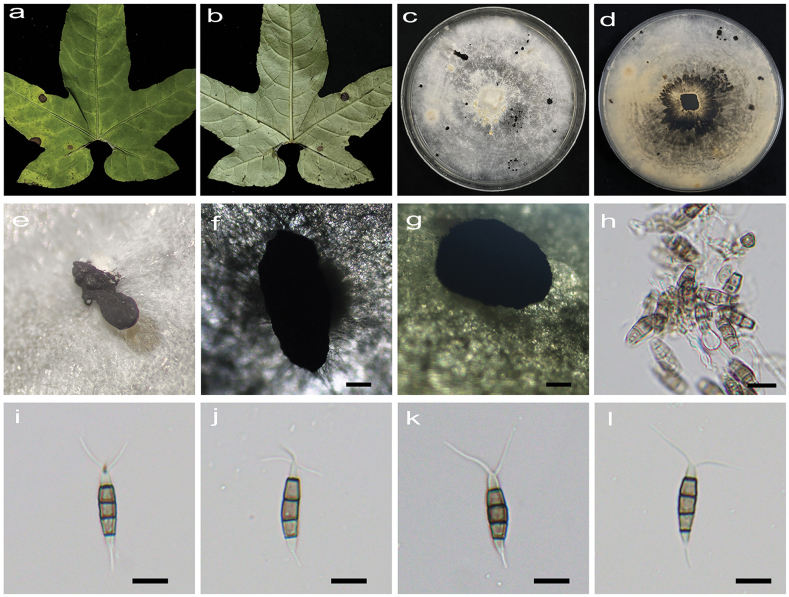
*Pestalotiopsishederae* (HJAUP C1638.221, ex-type) **a, b** leaf of host plant (front and reverse) **c, d** culture on PDA (front and reverse) **e–g** conidiomata **h** conidiogenous cells and conidia **i–k** conidia. Scale bars: 200 µm (**f, g**); 10 µm (**h–l**).

##### Etymology.

Referring to the host genus, *Hedera* from which it was collected.

##### Description.

Regular leaf spots, grey-brown in the center and darkening to black brown at the margins. Asexual morph on PDA: Conidiomata acervular, globose, 660–1570 μm diam., solitary or aggregated in clusters, black. Conidiophores indistinct and reduced to conidiogenous cells. Conidiogenous cells hyaline, smooth, cylindrical to ampulliform. Conidia fusiform, straight or slightly curved, 15.8–22.4 × 4.9–6.3 μm (x̄ = 18.0 × 5.7 μm, n = 50), 4-septate, slightly constricted at the septa, basal cell conical, 3.1–5.3 μm (x̄ = 4 μm), hyaline or sometimes pale brown, smooth, thin-walled, with a single filiform appendage, unbranched, 3.4–5.9 μm (x̄ = 4.8 μm) long; three median cells doliiform to cylindrical, smooth, thick-walled, 11.1–15.5 μm (x̄ = 13.6 μm), pale brown to brown, concolorous, somewhat constricted at the septa, second cell from the base 3.7–5.3 μm (x̄ = 4.7 μm) long, third cell 4.3–5.6 μm (x̄ = 4.9 μm) long, fourth cell 4.2–5.6 μm (x̄ = 5 μm) long; apical cell conical to acute, hyaline, smooth, thin-walled, 3.3–5 µm (x̄ = 4.1 μm) long, with 2(–3) filiform appendages, arising from the apex of the apical cell each at a different point, unbranched, 10.8–19.6 µm (x̄ = 15.5 μm) long. Sexual morph not observed.

##### Culture characteristics.

Colonies on PDA grow fast, filamentous to circular, growing all over the Petri dish at 25 °C in darkness, regular edge, white, sparse aerial mycelium on the surface, forming black conidiomata with black conidial masses, and reverse pale orange or white at the margin, dark brown at the center.

##### Additional specimen examined.

China • Yunnan Province, Jinghong City, Menghan Town, Xishuangbanna Dai Nationality Garden, 22 June 2022, X.X. Luo. On diseased leaves of *Hederahelix*, paratype HJAUP M1638.222, living culture HJAUP C1638.222.

##### Note.

Two strains (HJAUP C1638.221 and HJAUP C1638.222) of *Pestalotiopsishederae* isolated from leaf spots of *Hederahelix* formed a distinct clade sister to *P.hydei* (MFLUCC 20–0135) with 94% ML/0.95 BI bootstrap support (Fig. 1). The ex-type strain HJAUP C1638.221 is closely related to *P.hydei* (MFLUCC 20–0135) and comparisons of their nucleotides showed 10 bp differences (1%, including two gaps) nucleotide differences in three loci, respectively. Moreover, *P.hederae* is morphologically distinguished from *P.hydei* Huanraluek & Jayaward., which has longer conidia (18–35 μm vs. 15.8–22.4 μm) with minutely verruculose three median cells and shorter apical appendages (3–12 µm vs. 10.8–19.6 µm) (Huanaluek et al. 2021).

#### ﻿ Pestalotiopsis machiliana

Taxon classificationFungiAmphisphaerialesSporocadaceae

X.X. Luo and Jian Ma sp. nov.

7691D2D7-B98A-515C-BF4A-17B02F18C037

Index Fungorum: IF902325

Fig. 8

##### Type.

China • Jiangxi Province, Jingdezhen City, Changjiang District, Jingdezhen Botanical Garden; on diseased leaves of *Machiluspauhoi*; 3 November 2022; X.X. Luo (holotype HJAUP M1790.221; ex-type living culture HJAUP C1790.221).

**Figure 8.**
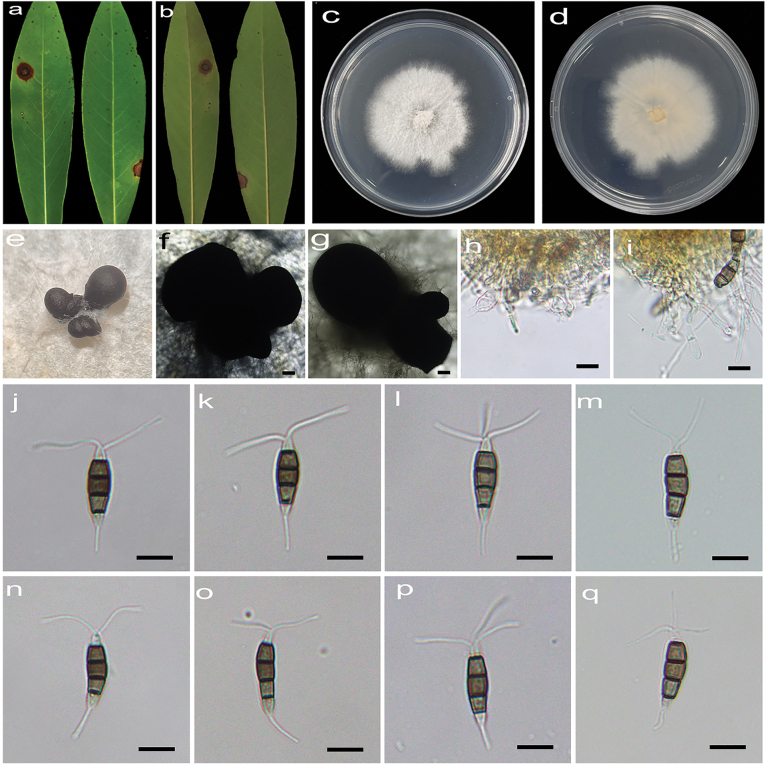
*Pestalotiopsismachiliana* (HJAUP C1790.221, ex-type) **a, b** leaf of host plant (front and reverse) **c, d** culture on PDA (front and reverse) **e–g** conidiomata **h, i** conidiogenous cells and conidia **j–q** conidia. Scale bars: 200 µm (**f, g**); 10 µm (**h–q**).

##### Etymology.

Referring to the host genus, *Machilus* from which it was collected.

##### Description.

Regular leaf spots, wheat in the center, a black stripe ring in the middle and dark brown at the margin. Asexual morph on PDA: Conidiomata acervular, globose, 646–1584 μm diam., solitary or aggregated in clusters, black. Conidiophores indistinct and reduced to conidiogenous cells. Conidiogenous cells hyaline, smooth, cylindrical to ampulliform. Conidia fusiform, straight or slightly curved, 18.6–27.2 × 5.6–7.4 μm (x̄ = 22.5 × 6.5 μm, n = 50), 4-septate, slightly constricted at the septa; basal cell conical, 3–5.2 μm (x̄ = 3.9 μm), hyaline or sometimes pale brown, smooth, thin-walled, with a single filiform appendage, unbranched, 4.5–10.2 μm (x̄ = 8.1 μm) long; three median cells doliiform to cylindrical, smooth, 12.5–17.3 μm (x̄ = 14.7 μm), concolorous, brown, somewhat constricted at the septa, second cell from the base 3.6–6.7 µm (x̄ = 5.0 μm) long, third cell 3.8−5.5 µm (x̄ = 4.6 μm) long, fourth cell 4.1–6.4 µm (x̄ = 4.9 μm) long; apical cell conical to acute, hyaline, smooth, thin-walled, 3–4.8 µm (x̄ = 3.9 μm) long, with 2–3 filiform appendages, arising from the apex of the apical cell each at a different point, unbranched, 12.9–22.5 µm (x̄ = 14.7 μm) long. Sexual morph not observed.

##### Culture characteristics.

Colonies on PDA grow fast, reaching 47–53 mm diam. after 5 days at 25 °C in darkness, white, with flocculent mycelium and entire edge, forming black conidiomata, and reverse buff.

##### Additional specimens examined.

China, Jiangxi Province, Jingdezhen City • Changjiang District, Jingdezhen Botanical Garden, 3 November 2022, X.X. Luo. On diseased leaves of *Machiluspauhoi*, paratype HJAUP M1790.222, living culture HJAUP C1790.222 • Fuliang County, Jingdezhen National Forest Park, 2 November 2022, X.X. Luo, on diseased leaves of *Rhododendronsimsii*, paratype HJAUP M1704.221, living culture HJAUP C1704.221; on diseased leaves of *Rhododendronsimsii*, paratype HJAUP M1704.222, living culture HJAUP C1704.222; on diseased leaves of *Rhododendronsimsii*, paratype HJAUP M1704.223, living culture HJAUP C1704.223.

##### Note.

Five strains (HJAUP C1790.221, HJAUP C1790.222, HJAUP C1704.221, HJAUP C1704.222 and HJAUP C1704.223) of *Pestalotiopsismachiliana* isolated from leaf spots of *Machiluspauhoi* clustered as a sister taxon to *P.chamaeropis* (CFCC 54977, CFCC 55023, CFCC 55019 and CFCC 55122) with 99% ML/0.97 BI bootstrap support (Fig. 1). The ex-type strain HJAUP C1790.221 is closely related to *P.chamaeropis* (CBS 186.71) and comparisons of their nucleotides showed 8 bp differences (1%, including one gap) nucleotide differences in three loci. Moreover, *P.machiliana* is morphologically distinguished from *P.chamaeropis* Maharachch., K.D. Hyde & Crous, which has minutely verruculose, wider conidia (7–9 μm vs. 5.6–7.4 μm) with longer basal cell (5–6.5 μm vs. 3–5.2 μm) and apical cell (4–6 µm vs. 3–4.8 µm) (Maharachchikumbura et al. 2014).

#### ﻿ Pestalotiopsis mangifericola

Taxon classificationFungiAmphisphaerialesSporocadaceae

X.X. Luo & Jian Ma sp. nov.

9968C0A5-3787-58FE-8CC0-A0E7A31EEF16

Index Fungorum: IF902326

Fig. 9

##### Type.

China • Yunnan Province, Xishuangbanna Dai Autonomous Prefecture, Mengla County, Menglun Town, Tropical Botanical Garden, on diseased leaves of *Mangiferaindica*, 23 June 2022, X.X. Luo (holotype HJAUP M1639.221; ex-type living culture HJAUP C1639.221).

**Figure 9.**
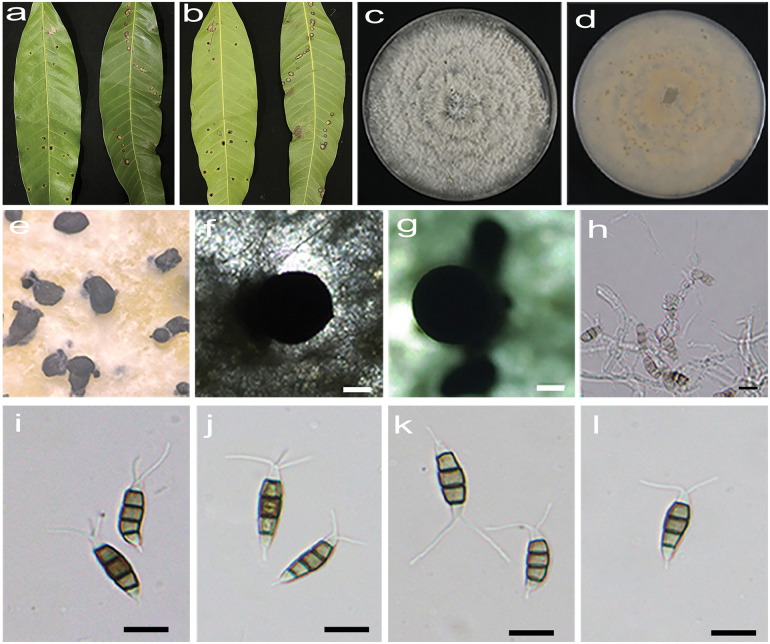
*Pestalotiopsismangifericola* (HJAUP C1639.221, ex-type) **a, b** leaf of host plant (front and reverse) **c, d** culture on PDA (front and reverse) **e–g** conidiomata **h** conidiogenous cells and conidia **i–l** conidia. Scale bars: 200 µm (**f**, **g**); 10 µm (**h**–**l**).

##### Etymology.

Referring to the host genus, *Mangifera* from which it was collected.

##### Description.

Regular leaf spots, initially brown with a yellowish halo around the edges, later yellowish-white center with black edges. Asexual morph on PDA: Conidiomata acervular, subglobular, 426–786 μm diam, solitary or aggregated in clusters, black. Conidiophores indistinct and reduced to conidiogenous cells. Conidiogenous cells hyaline, smooth, cylindrical to ampulliform. Conidia fusiform, straight or slightly curved, 13.5–18 × 4.7–6 μm (x̄ = 15.2 × 5.4 μm, n = 50), 4-septate, slightly constricted at the septa; basal cell conical, 2.9–4.4 μm (x̄ = 3.6 μm), hyaline or sometimes pale brown, smooth, thin-walled, with a single filiform appendage, unbranched, 3.1–5.5 μm (x̄ = 4.2μm) long; three median cells doliiform to cylindrical, smooth, 10.8–12.3 μm (x̄ = 11.5 μm), concolorous or sometimes darker at the central cell or the two upper cells, somewhat constricted at the septa, second cell from the base pale brown, 3.3–4.6 µm (x̄ = 3.9 μm) long, third cell pale brown to brown, 3.6–4.5 µm (x̄ = 3.9 μm) long, fourth cell pale to medium brown, 3.5–5.1 µm (x̄ = 4.2 μm) long; apical cell conical to acute, hyaline, smooth, thin-walled, 2.5–4 µm (x̄ = 3.1 μm) long, with 2–3 filiform appendages, arising from the apical crest, unbranched, 7.2–11.6 µm (x̄ = 9.8 μm) long. Sexual morph not observed.

##### Culture characteristics.

Colonies on PDA grow fast, filamentous to circular, growing all over the Petri dish (d = 8.5 cm) after 2 weeks at 25 °C in darkness, white, with flocculent aerial mycelium and entire edge, forming black conidiomata, and reverse pale orange.

##### Additional specimen examined.

China • Yunnan Province, Xishuangbanna Dai Autonomous Prefecture, Mengla County, Menglun Town, Tropical Botanical Garden, 23 June 2022, X.X. Luo. On diseased leaves of *Mangiferaindica*, paratype HJAUP M1639.222, living culture HJAUP C1639.222.

##### Note.

Two strains (HJAUP C1639.221 and HJAUP C1639.222) of *Pestalotiopsismangifericola* isolated from leaf spots of *Mangiferaindica* formed a distinct clade sister to *P.adusta* (MFLUCC 10–146 and ICMP 6088) with 100% ML/0.90 BI bootstrap support (Fig. 1). The ex-type strain HJAUP C1639.221 is closely related to *P.adusta* (ICMP 6088) and comparisons of their nucleotides showed 4 bp differences (1%, including one gap) nucleotide differences in three loci. Moreover, *P.mangifericola* is morphologically distinguished from *P.adusta* (Ellis & Everh.) Steyaert in its smaller conidia (13.5–18 × 4.7–6 μm vs. 16–22 × 5–7 μm) with shorter three median cells (10.8–12.3 μm vs. 12–15 μm) (Steyaert 1953; Maharachchikumbura et al. 2012).

## ﻿Discussion

The establishment of *Pestalotiopsis* was based on morphological studies. Members in the genus mainly occur in the asexual morph, and only 12 species have been linked with the sexual morphs (Maharachchikumbura et al. 2011). The generic concept of *Pestalotiopsis* is based on the characteristics of asexual morph and is mainly characterized by fusiform conidia and three pigmented median cells, each consisting of a hyaline basal cell and a hyaline apical cell with one or more simple or branched appendages (Steyaert 1949; Maharachchikumbura et al. 2014). These characters separate *Pestalotiopsis* from *Pestalotia* De Not. (with 6-celled conidia) and *Truncatella* Steyaert (with 4-celled conidia). Subsequently, Maharachchikumbura et al. (2014) revisited the genus *Pestalotiopsis* based on molecular evidence and the differences in the median cells of the conidia and proposed two segregated genera including *Neopestalotiopsis* and *Pseudopestalotiopsis*. Senanayake et al. (2015) treated *Pestalotiopsis*, *Pseudopestalotiopsis*, *Neopestalotiopsis* and other four genera in a new family, Pestalotiopsidaceae Maharachch. & K.D. Hyde, based on morphological similarities and sequence analysis.

To date, about 437 epithets for *Pestalotiopsis* have been listed in Index Fungorum (Index Fungorum 2024), but many species were introduced only based on morphological studies, and the excessive overlap of conidial features makes it difficult to identify *Pestalotiopsis* species only by morphology. Thus, there is presently a strong tendency to evaluate or clarify the taxonomic placements and phylogenetic relationships of *Pestalotiopsis* species by molecular methods. Maharachchikumbura et al. (2014) analyzed ten gene regions to resolve the bound species in *Neopestalotiopsis* and *Pestalotiopsis*, and finally screened three most applicable regions (ITS, *tef1-α*, and *tub2*). Since then, the number of *Pestalotiopsis* species is constantly being excavated and steadily increasing, and all described *Pestalotiopsis* species were identified based on the combined analyses of these three loci except for *P.sequoia*, *P.bulbophylli* and *P.chiaroscuro* using LSU, ITS, *tef1-α* and *tub2* (Hyde et al. 2016; Wang et al. 2017; Crous et al. 2022). Our BLASTn analyses of these sequences showed a high similarity in some *Pestalotiopsis* species, such as ITS, *tef1-α* and *tub2* of *P.ficicrescens* (MZ477311, MZ868328 and MZ868301) (Hyde et al. 2023) were 99.62, 99.79 and 98.56% similar to *P.biciliata* (KM199308, KM199505 and KM199399) (Maharachchikumbura et al. 2014); *P.taxicola* (OQ626673, OQ714338 and OQ714333) (Wang et al. 2024) were 100%, 99.25% and 100% similar to *P.unicolor* (JX398998, JX399063 and JX399029) (Maharachchikumbura et al. 2012); *P.linguae* (OP094104, OP186110 and OP186108) (Li et al. 2023) were 99.64, 98.74 and 98.26 similar to *P.parva* (KM199313, KM199509 and KM199405) (Maharachchikumbura et al. 2014), but the phylogenetic analyses conducted based on combined ITS, *tef1-α* and *tub2* sequence data showed more powerful resolution in delineating *Pestalotiopsis* species and higher bootstrap support values for most clades. Based on previous studies, we also conducted phylogenetic analyses using ITS, *tef1-α* and *tub2* sequences, and our newly obtained 24 strains nested within the genus *Pestalotiopsis* formed distinct clades with good support value, and can be recognized as eight new phylogenetic species.

*Pestalotiopsis* species are known worldwide as plant pathogens, endophytes, or saprophytes, and are widely distributed in tropical and temperate regions (Maharachchikumbura et al. 2014; Li et al. 2024; Zhao et al. 2024). In recent years, studies conducted on the alpha-taxonomy of *Pestalotiopsis* are mainly focused on the exploration of the hidden species diversity (Index Fungorum 2024). The leaves with typical spots diseased by *Pestalotiopsis* fungi are usually collected to obtain fungal isolates, and the strains are identified based on morphological and phylogenetic approaches, but little attention has been accorded to their pathogenicity. In our study, the survey of microfungi associated with plant diseased leaves from terrestrial habitat in Jiangxi and Yunnan provinces, China reveal eight new species, namely *P.alpinicola*, *P.camelliicola*, *P.cyclosora*, *P.eriobotryae*, *P.gardeniae*, *P.hederae*, *P.machiliana* and *P.mangifericola*. To our knowledge, *P.alpinicola*, *P.cyclosora* and *P.machiliana* are the first report that associated with the hosts *Alpiniazerumbet*, *Cyclosorusinterruptus*, *Machiluspauhoi* and *Microlepiamarginata*, which will broaden the host range of *Pestalotiopsis* species, and provide an important contribution to the field of plant pathology and fungal taxonomy. With the ongoing addition of *Pestalotiopsis* species, we believe that a comprehensive study of the genus will reveal more hidden *Pestalotiopsis* species from terrestrial plants.

## Supplementary Material

XML Treatment for Pestalotiopsis alpinicola

XML Treatment for Pestalotiopsis camelliicola

XML Treatment for Pestalotiopsis cyclosora

XML Treatment for Pestalotiopsis eriobotryae

XML Treatment for Pestalotiopsis gardeniae

XML Treatment for Pestalotiopsis hederae

XML Treatment for Pestalotiopsis machiliana

XML Treatment for Pestalotiopsis mangifericola

## ﻿Additional information

### Author ORCIDs

Ming-Gen Liao https://orcid.org/0009-0001-9537-1773

Rafael F. Castañeda-Ruiz https://orcid.org/0000-0003-0063-3265

Jian Ma https://orcid.org/0000-0001-9783-1860

Zhao-Huan Xu https://orcid.org/0009-0008-2641-7783

### Data availability

All of the data that support the findings of this study are available in the main text or Supplementary Information.

## Supplementary materials

Supplementary material 1 The concatenated ITS, tef1-α and tub2 sequences Data type fas https://binary.pensoft.net/file/1150715This dataset is made available under the Open Database License (http://opendatacommons.org/licenses/odbl/1.0/). The Open Database License (ODbL) is a license agreement intended to allow users to freely share, modify, and use this Dataset while maintaining this same freedom for others, provided that the original source and author(s) are credited.Xing-Xing Luo, Ming-Gen Liao, Kai Zhang, Rafael F. Castañeda-Ruíz, Jian Ma, Zhao-Huan Xu
